# Factors associated with sense of coherence in patients with malignant tumors: a systematic review and meta-analysis

**DOI:** 10.3389/fpsyg.2026.1774202

**Published:** 2026-04-09

**Authors:** Wenqing Xu, Jiayao Wang, Tianxue Deng, Tao Xu, Wen Xiao, Jiansu Liao, Yuqing Zhou, Danfeng Gu

**Affiliations:** 1 Affiliated Hospital of Jiangnan University, Wuxi, China; 2 Wuxi Medical College, Jiangnan University, Wuxi, China

**Keywords:** malignant tumors, sense of coherence, correlated factors, systematic review, meta-analysis

## Abstract

**Objectives:**

Sense of coherence (SOC) is a significant positive psychological resource that influences psychological outcomes and quality of life in patients with malignant tumors. This systematic review and meta-analysis aims to identify and synthesize factors associated with SOC in this population.

**Methods:**

A systematic search was carried out across 10 databases, from their inception to 12 October 2025, to identify studies exploring factors related to SOC in patients with malignant tumors. The Agency for Healthcare Research and Quality (AHRQ) Methodological Checklist and the Newcastle-Ottawa Scale (NOS) were used to evaluate the methodological quality of the included studies. Effect sizes were expressed as Pearson correlation coefficients (*r*). Heterogeneity was assessed using the *I*^2^ statistic and the *Q* test. Subgroup analyses and leave-one-out sensitivity analyses were conducted to explore sources of heterogeneity and assess result robustness.

**Results:**

Thirty-six studies were finally included, involving 11,007 participants. The systematic review revealed many factors associated with SOC, including time since disease remission, absence of self-reported functional limitations and disability, use of complementary therapies, sexual function, number of combined chronic conditions, severity of somatic symptoms, cancer-related fatigue, coping strategies, self-esteem, the level of resourcefulness, self-advocacy, self-care ability, post-traumatic growth, aging adaptation, meaning in life, family function, partner's SOC, Type C personality, death anxiety, stigma, perceived stress, perceived hospital and surgery-related stress, psychological distress, unmet needs, self-perceived burden, social alienation, disease perception, and partner's depression level. The meta-analysis showed several factors significantly associated with SOC, including depression [*r* = −0.65, 95% *CI* (−0.75, −0.51)], anxiety [*r* = −0.48, 95% *CI* (−0.61, −0.32)], acceptance of disability [*r* = 0.43, 95% *CI* (0.30, 0.54)], wellbeing [*r* = 0.44, 95% *CI* (0.24, 0.61)], perceived social support [*r* = 0.50, 95% *CI* (0.35, 0.62)], and quality of life [*r* = 0.41, 95% *CI* (0.29, 0.52)].

**Conclusions:**

In this systematic review and meta-analysis, 35 factors were identified as related to SOC in patients with malignant tumors. Clinical practice should be guided by positive psychology and consider various relevant factors to comprehensively explore the developmental mechanisms of SOC in patients with malignant tumors, thereby building more targeted interventions to improve SOC.

**Systematic review registration:**

https://www.crd.york.ac.uk/PROSPERO/view/CRD420251020931, identifier: CRD420251020931.

## 1 Introduction

Malignant tumors, a major issue in global public health, pose serious risks to human health and occupy a significant portion of the global burden of disease (Force et al., 2025). According to the International Agency for Research on Cancer (IARC) of the World Health Organization, approximately 20 million new cases of malignant tumors and 9.7 million deaths were reported globally in 2022 (Bray et al., 2024). By 2050, global new cases of malignant tumors are projected to increase by approximately 76.6% compared with 2022, with deaths expected to rise by about 89.7% (Bizuayehu et al., 2024). Patients with malignant tumors face a lot of uncertainty during diagnosis, treatment, and recovery, and they also have to deal with the physical stress of invasive treatments and the ongoing risk of recurrence and metastasis. These stressors compound to readily drive patients into persistent psychological distress, which can significantly affect both disease prognosis and overall quality of life (Wu et al., 2022).

With the rise of positive psychology in oncology, more scholars are focusing on the role of positive psychological resources for patients with malignant tumors. Salutogenesis, a health theory developed by Antonovsky, focuses on the origins of health rather than the causes of disease. Within this framework, sense of coherence (SOC) is a key concept that describes a person's general, lasting, and dynamic confidence in facing internal and external environmental stimuli, reflecting their overall feeling about life and stable way of thinking. Sense of coherence is made up of three elements: comprehensibility, manageability, and meaningfulness (Li and Zhang, 2023). In the context of malignant tumors, comprehensibility refers to how well patients understand cancer-related stress, which helps them better understand disease-related changes and gradually accept their illness. Manageability refers to patients' awareness of the internal and external resources available to them, which helps them feel more in control of treatment, symptoms, and daily life challenges. Meaningfulness refers to patients' perception of illness-related stress as a challenge worth facing, which gives them motivation to engage in treatment and recovery. A growing body of evidence indicates that SOC plays an important protective role in patients with malignant tumors. Specifically, a higher SOC has been shown to help patients understand and cope with their illness more effectively, thereby alleviating specific psychological burdens such as stress and depressive symptoms (Li et al., 2017). At the aggregate level, a meta-analysis by (Winger et al. 2016) further supports that SOC significantly buffers the psychological distress experienced by patients with cancer in the context of illness, highlighting its importance as a psychological protective factor. Also, a stronger SOC is often linked to higher resilience, helping patients recover more quickly in stressful situations and find meaning in their lives during illness, which can promote their overall quality of life (Li et al., 2025). Building on previous studies that have firmly established the significance of SOC, further exploring its formation and development holds important theoretical and clinical value.

The formation of SOC is shaped by the combined influence of multiple factors. A large number of empirical research suggest that its levels are closely associated with demographic characteristics (e.g., gender, age, and the level of income), disease and treatment factors (e.g., cancer-related fatigue), and psychosocial factors (e.g., depression and coping strategies). However, existing research findings remain inconsistent on some factors. For example, Zheng et al. (2023) found a negative association between age and SOC, along with a positive association between educational level and SOC; in contrast, Gustavsson-Lilius et al. (2007) reported the opposite pattern: age was positively associated with SOC, while educational level was negatively associated with SOC. By comparison, findings on other factors did not show clear directional differences, but the relevant evidence is scattered. Differences in study populations, measurement tools, and analytical methods are considerable, and systematic integration and clear explanation are still lacking. Furthermore, most relevant studies use small-sample, cross-sectional designs, which reduces the generalizability of their findings. Therefore, this study aims to synthesize existing evidence through a systematic review and meta-analysis to comprehensively evaluate the associations between SOC and demographic characteristics, disease- and treatment-related factors, psychosocial factors, and quality of life among patients with malignant tumors. In addition, potential sources of heterogeneity will be explored to obtain more reliable and generalizable conclusions, thereby providing evidence for future work to improve patients' SOC and to develop targeted interventions.

## 2 Methods

This study was registered in PROSPERO (Registration Number: CRD420251020931), and the report followed the PRISMA 2020 statement for systematic reviews and meta-analyses. The PRISMA 2020 checklist is provided in Appendix 1.

### 2.1 Search strategy

A systematic search was carried out in 10 databases: PubMed, Web of Science, Embase, PsycINFO, CINAHL, Cochrane Library, CNKI, WanFang, VIP, and SinoMed. This study used a combination of subject headings and free-text terms which were connected with the Boolean operators (AND, OR, NOT) to search for studies on factors related to SOC in patients with malignant tumors. In addition, a literature-tracing method was used to improve the completeness of the search. The searches were conducted in Chinese and English, covering the period from the creation of the databases to October 12, 2025. The main search terms comprised subject headings related to “sense of coherence” and “neoplasms,” together with corresponding free-text words. The complete search strategy is shown in the Supplementary Table 1.

### 2.2 Eligibility criteria

Inclusion criteria were as follows: (i) Study population: patients aged 18 years or older with pathologically confirmed malignant tumors; (ii) Study content: SOC was measured using reliable tools whose psychometric properties had been validated. At least one SOC-related factor, defined as any variable for which a statistical association with SOC was formally tested and reported, was assessed, and results reported a Pearson correlation coefficient or other statistics that could be converted to *r*, such as *t* or *F*-values; (iii) Study design: cross-sectional, cohort, and case-control studies. Exclusion criteria were as follows: (i) Duplicate publications using the same data; (ii) Studies with incomplete data, obvious mistakes (including, but not limited to, statistical method errors and discrepancies between textual descriptions and chart data), or data that could not be converted or used; (iii) Studies for which the full text was unavailable; (iv) Theses, conference papers, and case reports; (v) Studies with a methodology quality rating as low quality.

### 2.3 Study selection and data extraction

The literature was independently screened by two trained researchers. They extracted data and cross-checked their results, resolving any disagreements through discussion or consultation with a third researcher. The literature screening process was as follows: the identified studies were put into EndNote to remove duplicates. Researchers first checked the titles and abstracts independently according to the eligibility criteria, and then checked the full texts to decide the final list of included studies. The extracted data included first author, publication year, country, study design, sample size, cancer type, SOC assessment tool, SOC score, factors associated with SOC, and *r*, ρ, *t, F*, or *X*^2^ values. In longitudinal studies, the effect size from the final time point was solely extracted.

### 2.4 Assessment of quality

The 11-item checklist from the Agency for Healthcare Research and Quality (AHRQ) was used to check the methodological quality of the cross-sectional studies (Rostom et al., 2004). Scores of 0–3, 4–7, and 8–11 show low, moderate, and high quality, respectively (Zhao et al., 2025). The Newcastle-Ottawa Scale (NOS) was used to check the methodological quality of the cohort and case-control studies (Stang, 2010). Scores of 0–3, 4–6, and 7–9 show low, moderate, and high quality, respectively (Zhao et al., 2025). Quality assessment was conducted prior to final study inclusion. The included studies were independently evaluated by two researchers using quality assessment methods suitable for each study type. They cross-checked their results and resolved any disagreements through discussion or consultation with a third researcher.

### 2.5 Data analysis

The meta-analysis was carried out in R Studio (version 4.4.3) using the “metacor” package, with Pearson correlation coefficient (*r*) used as the effect size and its 95% confidence interval reported. If a study did not report the *r* but provided the ρ, *t, F* or χ^2^, these statistics were transformed into *r* according to the following formulas: $r\;=\;2\sin\left(\rho \frac{\Pi }{6}\right)\;$(Rupinski and Dunlap, 1996); $r\;=\sqrt{\frac{t^{2}}{t^{2}+df}}$ (Wolf, 1986); $r\;=\sqrt{\frac{F}{F+df}}$ (Wolf, 1986); *df* = *N*−2; $r\;=\sqrt{\frac{X^{2}}{N}}$ (Cramér, 1946). Here, the ρ, *t, F* or χ^2^ refer to the statistics reported in the original studies, and *df* represents the corresponding degrees of freedom. To improve the accuracy of the converted effect sizes, this study used the correction factor proposed by Poom and af Wåhlberg (2022). Specifically, each converted *r* value was multiplied by a formula-specific correction factor derived from Monte Carlo simulations to correct for systematic biases introduced by different conversion formulas. Because the *F* produced by analysis of variance (ANOVA) did not indicate the direction of *r*, the anticipated direction of *r* was determined by checking whether the group means consistently increased or decreased. When this tendency was not clear, the conversion was not carried out and the study was excluded. The *r* from each study were combined after undergoing *Fisher's z* transformation, which was used to make the effect-size distribution closer to normal and to improve the accuracy of the overall effect estimate (van Aert, 2023). After the pooled analysis was completed, the overall *z* was converted back to the *r* for reporting. Heterogeneity among the included studies was evaluated using the *I*^2^ statistic and the *Q* test. When *I*^2^ ≤ 50% and *P* ≥ 0.10, the studies were considered homogeneous, and a fixed-effects model was used; if not, a random-effects model was used. When there was high heterogeneity, subgroup analyses were done to see how study characteristics affected the effect sizes and to find the reasons for the heterogeneity. Using a leave-one-out method, sensitivity analyses were carried out to check each study's influence on the pooled effect size and the robustness of the outcomes. Considering that conducting a publication bias analysis is not meaningful when fewer than 10 studies are included, this study did not do such an analysis.

## 3 Results

### 3.1 Search results

**Figure 1:**
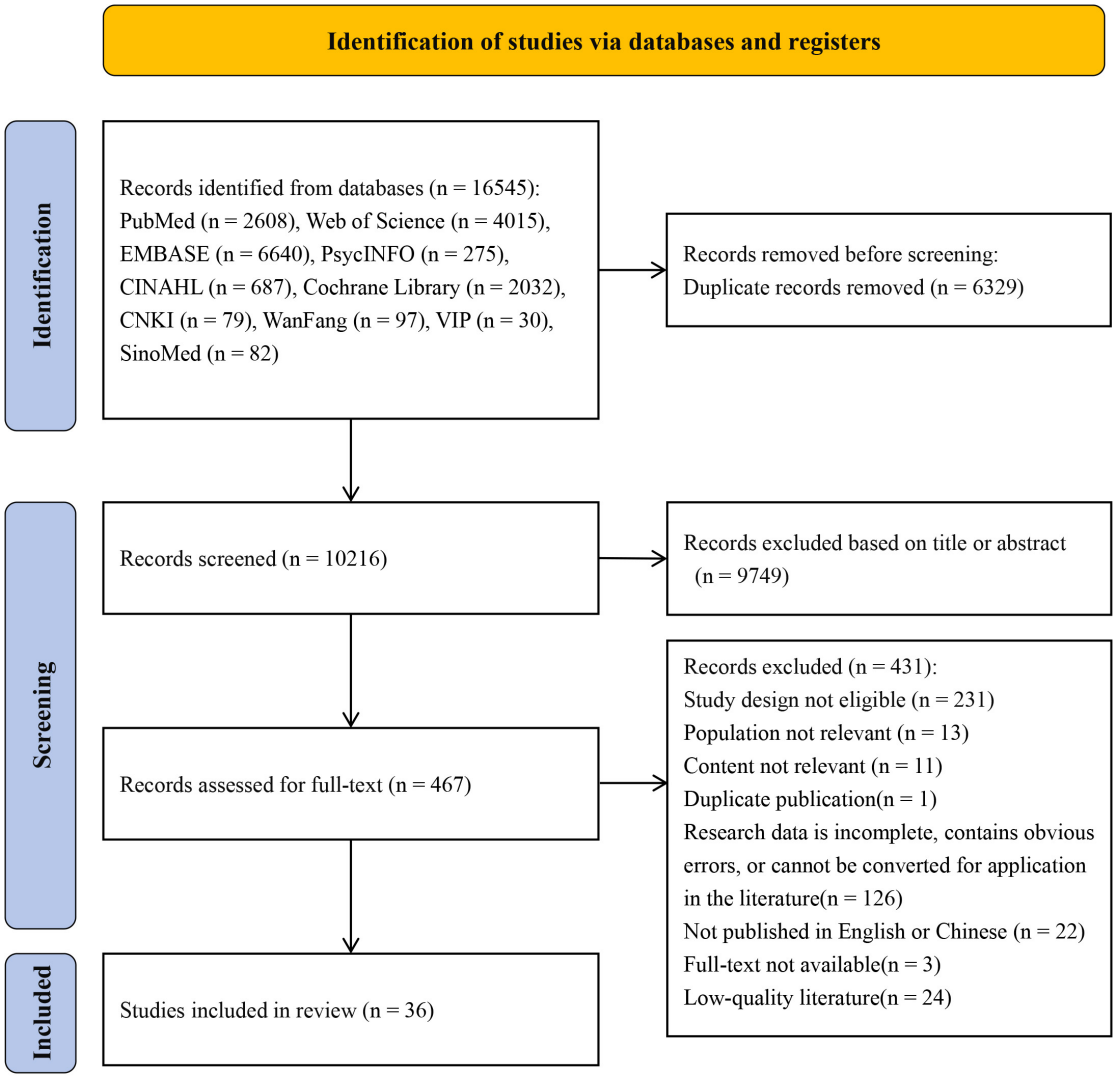
Flow diagram of the literature selection.

The initial search retrieved a total of 16,545 records, which was reduced to 10,216 after removing duplicates using EndNote. After an initial screening of titles and abstracts, a total of 467 articles underwent full-text re-screening, and 36 articles were finally included (Li et al., 2025, 2023; Zheng et al., 2023; Gustavsson-Lilius et al., 2007; Gu et al., 2024; Tang et al., 2017; Leonhart et al., 2017; Zhang et al., 2019; Guo et al., 2023; Liu et al., 2021; Wang et al., 2024, 2023; Wei et al., 2021; Liu and Li, 2019; He et al., 2023; Shen et al., 2016; Liu and Wang, 2015; Ge et al., 2019; Huang et al., 2023; Dadashi et al., 2024; Lashani et al., 2021; Zamanian et al., 2021, 2022; Aderhold et al., 2019; Festerling et al., 2023; Krampe et al., 2020; Vähäaho et al., 2021; Henoch et al., 2010, 2007; Bonacchi et al., 2014, 2019; Kim et al., 2021; Jabłoński et al., 2019; Asaba and Okawa, 2021; Paika et al., 2010; von Humboldt et al., 2019). The literature screening process was presented in Figure 1.

### 3.2 Characteristics of the included studies

A total of 36 articles (Li et al., 2025; Zheng et al., 2023; Gustavsson-Lilius et al., 2007; Gu et al., 2024; Tang et al., 2017; Leonhart et al., 2017; Zhang et al., 2019; Guo et al., 2023; Li et al., 2023; Liu et al., 2021; Wang et al., 2024, 2023; Wei et al., 2021; Liu and Li, 2019; He et al., 2023; Shen et al., 2016; Liu and Wang, 2015; Ge et al., 2019; Huang et al., 2023; Dadashi et al., 2024; Lashani et al., 2021; Zamanian et al., 2021, 2022; Aderhold et al., 2019; Festerling et al., 2023; Krampe et al., 2020; Vähäaho et al., 2021; Henoch et al., 2010, 2007; Bonacchi et al., 2014, 2019; Kim et al., 2021; Jabłoński et al., 2019; Asaba and Okawa, 2021; Paika et al., 2010; von Humboldt et al., 2019) were included in this study, with publication dates ranging from 2007 to 2025. The studies were conducted in 11 countries, including 18 from China (Li et al., 2025; Zheng et al., 2023; Gu et al., 2024; Tang et al., 2017; Leonhart et al., 2017; Zhang et al., 2019; Guo et al., 2023; Li et al., 2023; Liu et al., 2021; Wang et al., 2024, 2023; Wei et al., 2021; Liu and Li, 2019; He et al., 2023; Shen et al., 2016; Liu and Wang, 2015; Ge et al., 2019; Huang et al., 2023), 4 from Iran (Dadashi et al., 2024; Lashani et al., 2021; Zamanian et al., 2021, 2022), 3 from Germany (Aderhold et al., 2019; Festerling et al., 2023; Krampe et al., 2020), 2 from Finland (Gustavsson-Lilius et al., 2007; Vähäaho et al., 2021), 2 from Sweden (Henoch et al., 2010, 2007), 2 from Italy (Bonacchi et al., 2014, 2019), 1 from Korea (Kim et al., 2021), 1 from Poland (Jabłoński et al., 2019), 1 from Japan (Asaba and Okawa, 2021), 1 from Greece (Paika et al., 2010), and 1 from Portugal (von Humboldt et al., 2019). The included studies consisted of 32 cross-sectional studies (Li et al., 2025; Zheng et al., 2023; Gu et al., 2024; Tang et al., 2017; Leonhart et al., 2017; Zhang et al., 2019; Guo et al., 2023; Li et al., 2023; Liu et al., 2021; Wang et al., 2024, 2023; Wei et al., 2021; Liu and Li, 2019; He et al., 2023; Shen et al., 2016; Liu and Wang, 2015; Ge et al., 2019; Huang et al., 2023; Dadashi et al., 2024; Lashani et al., 2021; Zamanian et al., 2021, 2022; Aderhold et al., 2019; Festerling et al., 2023; Krampe et al., 2020; Henoch et al., 2010; Bonacchi et al., 2014, 2019; Kim et al., 2021; Asaba and Okawa, 2021; Paika et al., 2010; von Humboldt et al., 2019) and 4 cohort studies (Gustavsson-Lilius et al., 2007; Vähäaho et al., 2021; Henoch et al., 2007; Jabłoński et al., 2019). Among them, two studies (Zamanian et al., 2021, 2022) had completely overlapping samples. To avoid double counting, the correlation between SOC and quality of life was taken from only one of the studies (Zamanian et al., 2022); correlations with other variables (coping strategies, stigma) were included separately in the corresponding analyses (Zamanian et al., 2021, 2022). The combined sample size of included studies was 11,007. Specific characteristics are detailed in the Supplementary Table 2.

### 3.3 Quality of studies

The researchers reached a consensus on the methodological quality of the included studies, confirming that all satisfied the eligibility criteria for this study. The quality scores for cross-sectional studies, ranging from 4 to 8, were evaluated with the AHRQ checklist, while all cohort studies scored 4 points on the NOS scale. According to the scores described above, 1 study was evaluated as high quality (Lashani et al., 2021), and 35 studies were evaluated as moderate quality (Li et al., 2025; Zheng et al., 2023; Gustavsson-Lilius et al., 2007; Gu et al., 2024; Tang et al., 2017; Leonhart et al., 2017; Zhang et al., 2019; Guo et al., 2023; Li et al., 2023; Liu et al., 2021; Wang et al., 2024, 2023; Wei et al., 2021; Liu and Li, 2019; He et al., 2023; Shen et al., 2016; Liu and Wang, 2015; Ge et al., 2019; Huang et al., 2023; Dadashi et al., 2024; Zamanian et al., 2021, 2022; Aderhold et al., 2019; Festerling et al., 2023; Krampe et al., 2020; Vähäaho et al., 2021; Henoch et al., 2010, 2007; Bonacchi et al., 2014, 2019; Kim et al., 2021; Jabłoński et al., 2019; Asaba and Okawa, 2021; Paika et al., 2010; von Humboldt et al., 2019). Detailed quality assessment results are presented in the Supplementary Tables 3, 4.

### 3.4 Associated factors of SOC

#### 3.4.1 Demographic characteristics

##### 3.4.1.1 Gender

Two studies (Gustavsson-Lilius et al., 2007; Asaba and Okawa, 2021) reported the association between gender and SOC, and the pooled *r* showed a non-significant negative correlation between SOC and female gender [*r* = −0.01, 95% *CI* (−0.15, 0.14), *P* = 0.9389, *I*^2^= 0.0%] (Supplementary Figure 1).

##### 3.4.1.2 Age

Four studies (Zheng et al., 2023; Gustavsson-Lilius et al., 2007; Li et al., 2023; von Humboldt et al., 2019) reported the association between age and SOC, and the pooled *r* showed a non-significant negative correlation between SOC and age [*r* = −0.01, 95% *CI* (−0.21, 0.19), *P* = 0.9408, *I*^2^= 93.8%] (Supplementary Figure 2). Sensitivity analysis indicated that the study by Zheng et al. (2023) had a notable impact on the pooled effect size. After removing this study, the pooled *r* was 0.11 [95% *CI* (0.04, 0.19), *P* = 0.0034, *I*^2^= 17%] (Supplementary Figure 12), suggesting that this study could be the primary reason for heterogeneity.

##### 3.4.1.3 Level of income

Two studies (Zheng et al., 2023; von Humboldt et al., 2019) reported the association between the level of income and SOC, and the pooled *r* showed a non-significant positive correlation between SOC and the level of income [*r* = 0.54, 95% *CI* (−0.52, 0.95), *P* = 0.3147, *I*^2^= 99.7%] (Supplementary Figure 3).

##### 3.4.1.4 Educational level

Three studies (Zheng et al., 2023; Gustavsson-Lilius et al., 2007; von Humboldt et al., 2019) reported the association between educational level and SOC, and the pooled *r* showed a non-significant positive correlation between SOC and educational level [*r* = 0.34, 95% *CI* (−0.35, 0.79), *P* = 0.3322 , *I*^2^= 99.1%] (Supplementary Figure 4). Sensitivity analysis showed that the pooled *r* ranged from 0.01 to 0.53 after excluding any single study, with a consistent effect direction and no change in statistical significance. Heterogeneity remained high across all models (*I*^2^= 87.0%−99.5%), suggesting that no single study had a major effect on the overall results. Although the pooled effect size was relatively stable, the sources of heterogeneity require further investigation (Supplementary Figure 13).

#### 3.4.2 Disease- and treatment-related factors

The included studies reported several disease- and treatment-related factors associated with SOC. However, because only one study was available for each factor, meta-analysis was not performed and the findings were summarized descriptively. The results showed that time since disease remission (von Humboldt et al., 2019), absence of self-reported functional limitations and disability (von Humboldt et al., 2019), current or past use of complementary therapies (Bonacchi et al., 2014), and sexual function (Lashani et al., 2021) were significantly positively related to SOC. In contrast, number of combined chronic conditions (Zheng et al., 2023), severity of somatic symptoms (Leonhart et al., 2017), and cancer-related fatigue (Gu et al., 2024) were significantly negatively related to SOC.

#### 3.4.3 Psychosocial factors

##### 3.4.3.1 Depression

Six studies (Gustavsson-Lilius et al., 2007; Guo et al., 2023; Li et al., 2023; Wei et al., 2021; Aderhold et al., 2019; Kim et al., 2021) reported the association between depression and SOC, and the pooled *r* showed a significant negative correlation between SOC and depression [*r* = −0.65, 95% *CI* (−0.75, 20.51), *P* < 0.0001, *I*^2^= 97.7%] (Supplementary Figure 5). Sensitivity analysis showed that the pooled *r* ranged from −0.67 to −0.59 after excluding any single study, with a consistent effect direction and no change in statistical significance. After excluding the study by Guo et al. (2023), the *I*^2^ value decreased to below 50%, suggesting that this study could be the primary reason for heterogeneity (Supplementary Figure 14).

##### 3.4.3.2 Anxiety

Two studies (Gustavsson-Lilius et al., 2007; Li et al., 2023) reported the association between anxiety and SOC, and the pooled *r* showed a significant negative correlation between SOC and anxiety [*r* = 20.48, 95% *CI* (−0.61, −0.32), *P* < 0.0001, *I*^2^= 69.3%] (Supplementary Figure 6).

##### 3.4.3.3 Acceptance of disability

Two studies (Zhang et al., 2019; Liu et al., 2021) reported the association between acceptance of disability and SOC, and the pooled *r* showed a significant positive correlation between SOC and acceptance of disability [*r* = 0.43, 95% *CI* (0.30, 0.54), *P* < 0.0001, *I*^2^= 56.5%] (Supplementary Figure 7).

##### 3.4.3.4 Wellbeing

Four studies (Ge et al., 2019; Lashani et al., 2021; Krampe et al., 2020; von Humboldt et al., 2019) reported the association between wellbeing and SOC, and the pooled *r* showed a significant positive correlation between SOC and wellbeing [*r* = 0.44, 95% *CI* (0.24, 0.61), *P* < 0.0001, *I*^2^= 97.6%] (Supplementary Figure 8). As detailed in Supplementary Table 2, the wellbeing outcome comprised conceptually related constructs, including general wellbeing, mental wellbeing, and subjective wellbeing, which were synthesized as a broader overall wellbeing construct in the meta-analysis. Sensitivity analysis showed that the pooled *r* ranged from 0.39 to 0.54 after excluding any single study, with no change in effect direction and statistical significance. Regardless of which study was excluded, *I*^2^ stayed above 50%, showing that the heterogeneity was not caused by any single study. It was more likely related to differences in sample characteristics or methodologies among the included studies, indicating the need for further subgroup analyses to explore the sources of heterogeneity (Supplementary Figure 15).

##### 3.4.3.5 Resilience

Two studies (Li et al., 2025; Festerling et al., 2023) reported the association between resilience and SOC, and the pooled *r* showed a non-significant positive correlation between SOC and resilience [*r* = 0.79, 95% *CI* (−0.34, 0.99), *P* = 0.1389, *I*^2^= 99.4%] (Supplementary Figure 9).

##### 3.4.3.6 Perceived social support

Two studies (Li et al., 2023; Shen et al., 2016) reported the association between perceived social support and SOC, and the pooled *r* showed a significant positive correlation between SOC and perceived social support [*r* = 0.50, 95% *CI* (0.35, 0.62), *P* < 0.0001, *I*^2^= 78.4%] (Supplementary Figure 10).

##### 3.4.3.7 Coping strategies

Four studies (Liu and Li, 2019; Ge et al., 2019; Zamanian et al., 2021; Kim et al., 2021) reported the association between coping strategies and SOC. However, as none of these studies analyzed the correlation between total scores of coping strategies and SOC, and different assessment tools were used, only a descriptive analysis is provided here. Overall, positive coping strategies, such as active restructuring, planning, and acceptance, had a significant positive relationship with SOC, while negative coping strategies, like avoidance and giving in, had a significant negative relationship with SOC.

##### 3.4.3.8 Others

The included studies also reported other psychosocial factors. However, because each factor was included in only one study, a meta-analysis could not be performed. Therefore, only a descriptive analysis was conducted. The findings showed that self-esteem (Liu and Li, 2019), the level of resourcefulness (Wang et al., 2023), self-advocacy (He et al., 2023), self-care ability (Liu and Wang, 2015), post-traumatic growth (Shen et al., 2016), aging adaptation (von Humboldt et al., 2019), meaning in life (Li et al., 2025), family function (Wang et al., 2023), and partner's SOC (Gustavsson-Lilius et al., 2007) were significantly positively related to SOC. In contrast, Type C personality (Wei et al., 2021), death anxiety (Dadashi et al., 2024), stigma (Zamanian et al., 2022), perceived stress (Guo et al., 2023), perceived hospital and surgery-related stress (Krampe et al., 2020), psychological distress (Liu et al., 2021), unmet needs (Bonacchi et al., 2019), self-perceived burden (Wang et al., 2024), social alienation (Zheng et al., 2023), disease perception (Huang et al., 2023), and partner's depression level (Gustavsson-Lilius et al., 2007) were significantly negatively related to SOC.

#### 3.4.4 Quality of life

Eight studies (Tang et al., 2017; Wang et al., 2024; Zamanian et al., 2022; Vähäaho et al., 2021; Henoch et al., 2010, 2007; Kim et al., 2021; Jabłoński et al., 2019) reported the association between total quality of life and SOC, and the pooled *r* showed a significant positive correlation between SOC and quality of life [*r* = 0.41, 95% *CI* (0.29, 0.52), *P* < 0.0001, *I*^2^= 74.3%] (Supplementary Figure 11). Sensitivity analysis showed that the pooled *r* ranged from 0.39 to 0.46 after excluding any single study, with a consistent effect direction and no change in statistical significance. After removing the study by Jabłoński et al. (2019), the *I*^2^ decreased to below 50%, showing that this study could be the primary source of heterogeneity (Supplementary Figure 16). Additionally, two studies (Asaba and Okawa, 2021; Paika et al., 2010) reported correlations between SOC and various dimensions of quality of life, assessed using different quality of life measurement tools. Here, the findings are presented as descriptive Supplementary material: both studies consistently showed that patients with higher quality of life had higher levels of SOC.

### 3.5 Subgroup analyses

High-heterogeneity factors (*I*^2^ > 50%) were further analyzed in subgroups according to region, cancer type, SOC measurement tools, and related factor assessment tools to explore the reasons of heterogeneity. The findings of the subgroup analysis are shown in the Supplementary Table 5.

## 4 Discussion

This study aimed to identify the factors related to sense of coherence (SOC) in persons with malignant tumors. Thirty-six studies were finally included, reporting 40 relevant factors. Among these factors, 12 factors were included in qualitative analysis or quantitative synthesis due to having enough studies. The remaining 28 factors were summarized descriptively, as fewer than 2 studies were eligible for analysis. Thirty-five factors were identified as being associated with SOC, including 8 disease- and treatment-related factors, 26 psychological and social factors, and quality of life.

### 4.1 Demographic factors

A non-significant correlation between gender and SOC was found, with effect sizes close to 0, which suggests that, based on the current limited evidence, gender cannot yet be supported as an important factor associated with SOC. This differs from previous findings in cancer samples, which reported a negative correlation between female gender and SOC (Thomé and Hallberg, 2004). One possible explanation for this negative relation is that women may report more physical and mental problems and face stronger role conflicts, which could weaken their ability to comprehend and manage their illness and affect the construction of a sense of meaning (Miraglia Raineri et al., 2021). However, this combined effect was derived from only two studies and therefore had limited statistical power; as a result, the finding should be interpreted carefully.

A non-significant correlation between age and SOC was found, in line with earlier research conducted among cancer patients (Winger et al., 2016). In analyzing the underlying reasons, from a theoretical point of view, earlier research suggests that SOC gradually becomes stable around the age of 30, and shows relatively limited natural variation across age groups (Geyer, 1997). Therefore, the impact of age itself on SOC may be relatively weak; from a methodological point of view, the included studies showed differences in tumor type, disease stage, treatment setting, and the control of confounding variables, which may have introduced significant inter-study heterogeneity and weakened the age effect that might have appeared in single studies when they were pooled together. Given that the heterogeneity remained high even after subgroup analyses, this finding should be viewed with caution as unmeasured confounders might be at play.

A non-significant correlation between the level of income and SOC was found. However, existing research suggested that the income level may affect the SOC of cancer patients (Bargehr et al., 2023). Malignant tumors are often associated with high treatment costs, loss of income, and caregiving burdens, placing patients under great financial stress. In contrast, people with higher incomes, supported by better material security, access to resources, and stronger social support, are more likely to keep stable and manageable psychological experiences when coping with illness and its related pressures (Le et al., 2020). As a result, they tend to better understand their illness, feel more control during the coping process, and keep a sense of meaning in life during long-term adjustment. Because of limited information and high heterogeneity between studies, results about the association between income level and SOC remain tentative.

A non-significant correlation between educational level and SOC was found. It should be noted that this finding does not mean that educational level has no effect on SOC. In considering the underlying reasons, one possibility is that SOC is made up of three dimensions: comprehensibility, manageability, and meaningfulness. Educational level may mainly influence the parts that are related to cognition, making it difficult to detect significant differences in the overall SOC level. Secondly, the existential threat and intense emotional burden caused by a malignant tumor diagnosis may temporarily reduce the advantage in understanding information that people with higher education usually have Hong et al. (2022). However, this is only one possible reason and cannot fully explain the whole situation. Moreover, because only a few studies were included, further subgroup analyses based on region, cancer type, or SOC measurement tool could not be conducted. As a result, the high heterogeneity in this study remains unexplained and necessitates a cautious interpretation of this finding.

### 4.2 Disease- and treatment-related factors

This study summarized disease- and treatment-related factors associated with SOC among patients with malignant tumors, including time since disease remission, self-reported functional limitations and disability, number of combined chronic conditions, severity of somatic symptoms, cancer-related fatigue, use of complementary therapies, and sexual function. Among these, the number of combined chronic conditions, severity of somatic symptoms, and cancer-related fatigue were negatively associated with SOC. In contrast, time since disease remission, self-reported absence of functional limitations and disability, and the use of complementary therapies were positively associated with SOC. These findings agree with the salutogenic model of health, which posits that a higher disease burden continuously depletes an individual's general resistance resources and lower their SOC. In contrast, as more time passes after disease remission, and as physical function improves and people engage in health-promoting behaviors, these resources can be easier to accumulate, leading to higher SOC levels (Li and Zhang, 2023). Moreover, this study found that higher levels of overall sexual function were positively related to SOC. Research showed that sexual problems, which were common in cancer patients, were often overlooked. Treatments including surgery, radiotherapy, chemotherapy, and hormone therapy can harm sexual function, which may affect patients' mental health and their interpersonal relationships (Emery et al., 2022). Patients with higher levels of sexual function usually have more positive physical and mental experiences and stronger social support networks, which contribute to keeping and improving their SOC.

### 4.3 Psychosocial factors

A negative correlation between depression and SOC was found, consistent with previous meta-analyses on patients with chronic pain (Aguilar-Latorre et al., 2023). Patients with higher levels of depression often have negative cognitive biases, lower self-efficacy, and a weaker sense of meaning (Liu et al., 2023), which reduces their understanding of illness, their ability to use available resources, and their sense of meaning in life, and thus leads to a lower overall SOC. Subgroup analysis showed that different depression assessment tools could, in part, explain the high heterogeneity among studies. Different depression scales have differences in measurement content, item composition, sensitivity, and scoring methods. This may cause systematic bias in estimating depression levels, which can affect the size of the association between depression and SOC and increase heterogeneity between studies. This finding suggests that future research should improve consistency in the choice of depression measurement tools or use standardized procedures to improve the comparability of study results.

This study found a negative correlation between anxiety and SOC. This relationship may be related to reduced cognitive processing ability under high levels of anxiety. Previous studies have shown that people with higher anxiety tend to have poorer cognitive control, especially in emotional situations, with reduced ability to regulate attention and process information efficiently (Shen et al., 2025). In the context of illness, such cognitive control problems may cause patients to focus their attention more narrowly, making it difficult for them to understand and combine disease-related information in a timely and effective way (Bashian et al., 2025). As a result, patients may struggle to build a coherent cognitive framework of their illness, which reduces their ability to comprehend and manage the condition, ultimately lowering their overall SOC. Because the included studies provided limited information and showed high heterogeneity, the relationship between anxiety and SOC should be viewed carefully.

Acceptance of disability was found to be positively correlated with SOC. Cancer treatments, which often change patients' appearance and body functions, can lead to psychosocial stress by affecting body image; for example, colorectal cancer patients may need long-term stoma care, and breast cancer patients may have a mastectomy (Brederecke et al., 2021). If patients accept these changes and incorporate disability into their self-concept, they are more likely to achieve better comprehensibility and manageability in their psychological adaptation, which allows them to reconstruct meaning within their new physical condition and strengthens their confidence in life (Thornton and Lewis-Smith, 2023). However, the included studies provided limited information and showed high heterogeneity, necessitating a cautious view of the relationship between disability acceptance and SOC.

A positive correlation between wellbeing and SOC was found, in line with earlier studies among patients with coronary heart disease (Krok, 2022). High levels of wellbeing often come with more positive emotional experiences and greater flexibility in thinking, which make people understand illness and stress in an adaptive way (Karbalaie et al., 2021). Such a cognitive orientation helps individuals keep a coherent understanding and sense of meaning about their illness experience, and it also increases their perceived control and confidence when facing stressful situations. Subgroup analysis showed that differences in SOC assessment tools could explain the high heterogeneity among studies. This may be related to differences in the number of items, the focus of the measurement dimensions, and the scoring methods of the scales, suggesting that future cross-study comparisons or meta-analyses should pay attention to the consistency of tools or consider standardizing different scales to improve the comparability and reliability of results.

A non-significant correlation between resilience and SOC was found, which is different from the finding of Hinz et al. (2023), who reported a positive association between the two variables. The positive correlation may be explained by the view that resilience is usually seen as an important personality trait, helping people keep mental stability when facing strong stress or uncertainty and recover quickly from difficulties (Schellekens et al., 2024). Patients with higher resilience usually have better emotional regulation and tend to use more active coping strategies, which helps them feel more confident in managing their illness (Macía et al., 2020). Because the included studies provided limited information and showed high heterogeneity, the relationship between resilience and SOC should be approached with a degree of caution.

Perceived social support was found to be positively correlated with SOC, which is in line with earlier findings in lung cancer patients (Wang et al., 2024). A higher level of perceived social support not only provides emotional comfort, guidance, and practical assistance to patients with malignant tumors, but also increases their awareness of available resources for coping with stress and helps them develop a more positive sense of meaning during their illness and treatment (Siao et al., 2024). However, given that only two studies were included and high heterogeneity existed between them, this finding should be viewed cautiously.

This study systematically reviewed the relationship between coping strategies and SOC, and found that positive coping strategies, including confrontation, planning, acceptance, positive reframing, and the use of emotional and instrumental support, were related to higher SOC, while negative coping strategies such as avoidance, resignation, behavioral disengagement, venting, and self-blame were related to lower SOC. Further analysis of different coping styles showed that problem-focused approaches like confrontation and planning prompt patients to identify stressors actively and take action, improving their perception of predictability and controllability over the disease process (Stanisławski, 2019). Acceptance and positive reframing, meanwhile, adjust persons' cognitive evaluations of disease events, reducing resistance to and denial of uncontrollable outcomes while promoting integration of the illness experience and reconstruction of meaning (Purc-Stephenson and Edwards, 2024). The use of emotional and instrumental support strengthens individuals' awareness of available external resources, alleviates the subjective burden of disease-related stress, and thereby enhances confidence in coping with health challenges (Peng et al., 2023; Ding et al., 2024). Conversely, negative coping strategies such as avoidance, resignation, behavioral disengagement, venting, and self-blame can keep patients feeling helpless, confused, and out of control, which reduces their ability to stay mentally stable and balanced (Zhu et al., 2024; Dev et al., 2023). However, all studies included in this analysis were cross-sectional, so their ability to make causal inferences is limited, and it is difficult to explain the potential mechanisms of this association. Future studies should employ longitudinal or interventional approaches to test the causal links between these factors.

This study identified other psychosocial factors related to SOC in patients with malignant tumors. Specifically, patients' self-esteem, resourcefulness, self-advocacy, self-care ability, post-traumatic growth, adaptation to aging, meaning in life, family function, and their partner's SOC were all positively related to SOC. In contrast, patients' type C personality, death anxiety, stigma, perceived stress, perceived hospital and surgery-related stress, psychological distress, unmet needs, self-perceived burden, social alienation, disease perception, and their partner's depression level were negatively related to SOC. These findings suggest that illness-related negative thoughts and emotions may reduce patients' SOC, while increasing positive psychosocial resources and improving self-regulatory abilities could further enhance it. It is important to note that SOC is not only based on the patient's own psychological resources, but is also strongly influenced by their partner and family system. Family support quality, emotional alignment in close relationships, and effective family functioning may act as buffers in patients' coping with the stresses of illness (van Roij et al., 2022; Wang et al., 2021). Therefore, while focusing on the patient's own psychological adjustment, it is also important to pay attention to the psychosocial wellbeing of their partner and family. Improving the quality of family interactions and strengthening spousal support can further promote the patient's SOC.

### 4.4 Quality of life

A positive correlation between SOC and quality of life was found, which is consistent with a earlier systematic review in cancer patients (Farajzadegan et al., 2021). Subgroup analysis results showed that differences in regions and SOC assessment tools could explain the high heterogeneity between studies, which suggests that cultural background and SOC assessment methods may play an important role in SOC and its connection with quality of life. Cultural values, social support systems, and health belief frameworks in different regions play a key role in shaping a person's sense of comprehensibility, manageability, and meaningfulness, which may cause differences in SOC scores across cross-cultural contexts. Moreover, structural differences in SOC scales, such as the number of items, measurement focus, and scoring methods, may reduce the comparability of results across studies and increased overall heterogeneity. The main direction of the positive association between SOC and quality of life remains to be clarified. While the mainstream view tends to consider SOC as an antecedent of quality of life (Vähäaho et al., 2021), some researchers have suggested that the two may influence each other during the adaptation process (Alcantara et al., 2020; Dziuba et al., 2021).

## 5 Limitations

However, several limitations should be noted in this study. First, because of the small number of included studies and the high heterogeneity among them, it was difficult to identify the sources of heterogeneity for some factors through subgroup analyses; sensitivity analyses also showed that the pooled effect for age was unstable, as the direction of the association changed during the leave-one-out analysis. Second, as fewer than ten studies were available for each factor, a reliable assessment of publication bias could not be conducted, which may limit the comprehensiveness of the results and the adequacy of the conclusions. Third, most studies were cross-sectional, which were unable to identify causal relationships between the factors mentioned above and SOC. Finally, half of the studies were carried out in China, which could introduce some cultural bias.

## 6 Conclusions

This study synthesized existing evidence on factors related to sense of coherence (SOC) in persons with malignant tumors. The findings suggest that psychosocial factors and quality of life are most strongly linked to patients' SOC and may play a key role in its formation, maintenance, and development. In contrast, studies on demographic factors and disease- and treatment-related factors are limited in number and show high heterogeneity. Their associations with SOC still lack consistent evidence and need to be further verified in future studies. Based on the results of this study, SOC assessment should be included in routine psychological screening for patients with malignant tumors to identify those with low SOC who may be at high risk of psychological distress and reduced quality of life. Psychosocial factors may be an important and modifiable basis for SOC. Accordingly, targeted intervention strategies could be developed and evaluated in future research to improve patients' adjustment to illness and their sense of control over their lives by strengthening positive psychological resources and social support networks. Furthermore, future research should conduct larger-scale, higher-quality longitudinal and interventional studies to explore the causal mechanisms between these factors and SOC, thereby providing the empirical basis for developing more targeted and effective psychological intervention strategies.

## Data Availability

The original contributions presented in the study are included in the article/Supplementary material, further inquiries can be directed to the corresponding author.
