# Supplementary material for: Factors associated with sense of coherence in patients with malignant tumors: a systematic review and meta-analysis
Source: Front Psychol. 2026 Apr 9;17:1774202. doi: 10.3389/fpsyg.2026.1774202 (PMC13102640; doi:10.3389/fpsyg.2026.1774202)
Supplement: Supplementary file 1 [file Data_Sheet_1.docx]

**Table 1: Searching strategies.**

| Database | Searching strategies | Results |
| --- | --- | --- |
| PubMed | #1 (((((((((Neoplasms[MeSH Terms]) OR (Tumor*[Title/Abstract])) OR (Tumour*[Title/Abstract])) OR (Neoplas*[Title/Abstract])) OR (Cancer*[Title/Abstract])) OR (Malignanc*[Title/Abstract])) OR (oncolog*[Title/Abstract])) OR (Neoplasm*, Malignant[Title/Abstract])) OR (Malignant Neoplasm*[Title/Abstract])) OR (CA[Title/Abstract])  #2 (((((((((((((((Carcinoma[MeSH Terms]) OR (Carcinoma*[Title/Abstract])) OR (Epithelioma*[Title/Abstract])) OR (Epithelial Neoplasm*, Malignant[Title/Abstract])) OR (Malignant Epithelial Neoplasm*[Title/Abstract])) OR (Neoplasm*, Malignant Epithelial[Title/Abstract])) OR (Epithelial Tumor*, Malignant[Title/Abstract])) OR (Malignant Epithelial Tumor*[Title/Abstract])) OR (Tumor*, Malignant Epithelial[Title/Abstract])) OR (Carcinoma, Anaplastic[Title/Abstract])) OR (Anaplastic Carcinoma*[Title/Abstract])) OR (Carcinoma, Spindle-Cell[Title/Abstract])) OR (Carcinoma, Spindle Cell[Title/Abstract])) OR (Spindle-Cell Carcinoma*[Title/Abstract])) OR (Carcinoma, Undifferentiated[Title/Abstract])) OR (Undifferentiated Carcinoma*[Title/Abstract])  #3 #1 OR #2  #4 (((((((((Sense of Coherence[MeSH Terms]) OR (sense of coherence[Title/Abstract])) OR (Coherence Sense[Title/Abstract])) OR (Salutogenes*[Title/Abstract])) OR (salutogenic*[Title/Abstract])) OR (salutogenesis[Title/Abstract])) OR (generalised resistance resource*[Title/Abstract])) OR (health asset*[Title/Abstract])) OR (SOC[Title/Abstract])) OR (Antonovsky[Title/Abstract])  #5 #3 AND #4 | 2608 |
| Web of science | **#1 ((((((((TS=(Tumor* )) OR TS=(Tumour* )) OR TS=(Neoplas* )) OR TS=(Cancer* )) OR TS=(Malignanc* )) OR TS=( oncolog* )) OR TS=(Neoplasm*, Malignant)) OR TS=(Malignant Neoplasm* )) OR TS=(CA)**  **#2 ((((((((((((((TS=(Carcinoma*)) OR TS=(Epithelioma* )) OR TS=(Epithelial Neoplasm*, Malignant )) OR TS=(Malignant Epithelial Neoplasm* )) OR TS=(Neoplasm*, Malignant Epithelial )) OR TS=(Epithelial Tumor*, Malignant )) OR TS=(Malignant Epithelial Tumor* )) OR TS=(Tumor*, Malignant Epithelial )) OR TS=(Carcinoma, Anaplastic )) OR TS=(Anaplastic Carcinoma* )) OR TS=(Carcinoma, Spindle-Cell )) OR TS=(Carcinoma, Spindle Cell )) OR TS=(Spindle-Cell Carcinoma* )) OR TS=( Carcinoma, Undifferentiated )) OR TS=(Undifferentiated Carcinoma*)**  **#3 #1 OR #2** #4 **((((((((TS=(sense of coherence )) OR TS=( Coherence Sense )) OR TS=(Salutogenes* )) OR TS=(salutogenic* )) OR TS=( salutogenesis )) OR TS=(generalised resistance resource* )) OR TS=( health asset* )) OR TS=(SOC )) OR TS=(Antonovsky)**  **#5 #3 AND #4** | 4015 |
| Embase | #1 ('neoplasm'/exp OR 'neoplasm') AND [embase]/lim  #2 ('acral tumo?r':ti,ab,kw OR 'neoplas*':ti,ab,kw OR 'neoplastic disease':ti,ab,kw OR 'neoplastic entity':ti,ab,kw OR 'neoplastic mass':ti,ab,kw OR 'tumor*':ti,ab,kw OR 'tumor* entity':ti,ab,kw OR 'tumor* mass':ti,ab,kw OR 'tumour*':ti,ab,kw OR 'tumour* entity':ti,ab,kw OR 'tumour* mass':ti,ab,kw OR 'malignanc*':ti,ab,kw OR 'oncolog*':ti,ab,kw OR 'ca':ti,ab,kw) AND [embase]/lim  #3 ('carcinoma'/exp OR 'carcinoma') AND [embase]/lim  #4 **('carcinoma 63':ti,ab,kw OR 'carcinoma, krebs 2':ti,ab,kw OR 'carcinoma, scirrhous':ti,ab,kw OR 'epithelial carcinoma':ti,ab,kw OR 'epithelial malignant tumo?r':ti,ab,kw OR 'internal carcinoma':ti,ab,kw OR 'malignant epithelial tumo?r':ti,ab,kw OR 'microcarcinoma':ti,ab,kw OR 'neoplasm, malignant epithelial':ti,ab,kw OR 'neoplasms, ductal, lobular, and medullary':ti,ab,kw OR 'primary carcinoma':ti,ab,kw OR 'carcinoma':ti,ab,kw) AND [embase]/lim**  #5 ('malignant neoplasm'/exp OR 'malignant neoplasm') AND [embase]/lim  #6 ('cancer*':ti,ab,kw OR 'malignant neoplas*':ti,ab,kw OR 'malignant neoplastic disease':ti,ab,kw OR 'malignant tumo?r':ti,ab,kw OR 'neoplas*, malignant':ti,ab,kw OR 'neoplas* malignancy':ti,ab,kw OR 'oncologic* malignancy':ti,ab,kw OR 'tumor* malignancy':ti,ab,kw OR 'tumo?r, malignant':ti,ab,kw) AND [embase]/lim  #7 #1 OR #2 OR #3 OR #4 OR #5 OR #6  #8 ('sense of coherence'/exp OR 'sense of coherence') AND [embase]/lim  #9 ('sense of coherence':ti,ab,kw OR 'coherence sense':ti,ab,kw OR 'soc':ti,ab,kw OR 'salutogenes*':ti,ab,kw OR 'salutogenic*':ti,ab,kw OR 'salutogenesis':ti,ab,kw OR 'generalised resistance resource*':ti,ab,kw OR 'health asset*':ti,ab,kw OR 'antonovsky':ti,ab,kw) AND [embase]/lim  #10 #8 OR #9  #11 #7 AND #10 | 6640 |
| PsycINFO | **S1 TI (Tumor* OR Tumour* OR Neoplas* OR Cancer* OR Malignanc* OR oncolog* OR Neoplasm*, Malignant OR Malignant Neoplasm* OR CA) OR AB (Tumor* OR Tumour* OR Neoplas* OR Cancer* OR Malignanc* OR oncolog* OR Neoplasm*, Malignant OR Malignant Neoplasm* OR CA) OR KW (Tumor* OR Tumour* OR Neoplas* OR Cancer* OR Malignanc* OR oncolog* OR Neoplasm*, Malignant OR Malignant Neoplasm* OR CA)**  **S2 TI (Carcinoma* OR Epithelioma* OR Epithelial Neoplasm*, Malignant OR Malignant Epithelial Neoplasm* OR Neoplasm*, Malignant Epithelial OR Epithelial Tumor*, Malignant OR Malignant Epithelial Tumor* OR Tumor*, Malignant Epithelial OR Carcinoma, Anaplastic OR Anaplastic Carcinoma* OR Carcinoma, Spindle-Cell OR Carcinoma, Spindle Cell OR Spindle-Cell Carcinoma* OR Carcinoma, Undifferentiated OR Undifferentiated Carcinoma*) OR AB (Carcinoma* OR Epithelioma* OR Epithelial Neoplasm*, Malignant OR Malignant Epithelial Neoplasm* OR Neoplasm*, Malignant Epithelial OR Epithelial Tumor*, Malignant OR Malignant Epithelial Tumor* OR Tumor*, Malignant Epithelial OR Carcinoma, Anaplastic OR Anaplastic Carcinoma* OR Carcinoma, Spindle-Cell OR Carcinoma, Spindle Cell OR Spindle-Cell Carcinoma* OR Carcinoma, Undifferentiated OR Undifferentiated Carcinoma*) OR KW (Carcinoma* OR Epithelioma* OR Epithelial Neoplasm*, Malignant OR Malignant Epithelial Neoplasm* OR Neoplasm*, Malignant Epithelial OR Epithelial Tumor*, Malignant OR Malignant Epithelial Tumor* OR Tumor*, Malignant Epithelial OR Carcinoma, Anaplastic OR Anaplastic Carcinoma* OR Carcinoma, Spindle-Cell OR Carcinoma, Spindle Cell OR Spindle-Cell Carcinoma* OR Carcinoma, Undifferentiated OR Undifferentiated Carcinoma*)**  **S3 S1 OR S2**  **S4 TI (sense of coherence OR Coherence Sense OR Salutogenes* OR salutogenic* OR salutogenesis OR generalised resistance resource* OR health asset* OR SOC OR Antonovsky) OR AB (sense of coherence OR Coherence Sense OR Salutogenes* OR salutogenic* OR salutogenesis OR generalised resistance resource* OR health asset* OR SOC OR Antonovsky) OR KW (sense of coherence OR Coherence Sense OR Salutogenes* OR salutogenic* OR salutogenesis OR generalised resistance resource* OR health asset* OR SOC OR Antonovsky)**  **S5 S3 AND S4** | 275 |
| CINAHL | S1 MH (MH "Neoplasms+") OR TI (Tumor* OR Tumour* OR Neoplas* OR Cancer* OR Malignanc* OR oncolog* OR Neoplasm*, Malignant OR Malignant Neoplasm* OR CA) OR AB (Tumor* OR Tumour* OR Neoplas* OR Cancer* OR Malignanc* OR oncolog* OR Neoplasm*, Malignant OR Malignant Neoplasm* OR CA)  S2 MH (MH "Carcinoma+") OR TI (Carcinoma* OR Epithelioma* OR Epithelial Neoplasm*, Malignant OR Malignant Epithelial Neoplasm* OR Neoplasm*, Malignant Epithelial OR Epithelial Tumor*, Malignant OR Malignant Epithelial Tumor* OR Tumor*, Malignant Epithelial OR Carcinoma, Anaplastic OR Anaplastic Carcinoma* OR Carcinoma, Spindle-Cell OR Carcinoma, Spindle Cell OR Spindle-Cell Carcinoma* OR Carcinoma, Undifferentiated OR Undifferentiated Carcinoma*) OR AB (Carcinoma* OR Epithelioma* OR Epithelial Neoplasm*, Malignant OR Malignant Epithelial Neoplasm* OR Neoplasm*, Malignant Epithelial OR Epithelial Tumor*, Malignant OR Malignant Epithelial Tumor* OR Tumor*, Malignant Epithelial OR Carcinoma, Anaplastic OR Anaplastic Carcinoma* OR Carcinoma, Spindle-Cell OR Carcinoma, Spindle Cell OR Spindle-Cell Carcinoma* OR Carcinoma, Undifferentiated OR Undifferentiated Carcinoma*)  S3 MM (MM "Sense of Coherence") OR TI (sense of coherence OR Coherence Sense OR Salutogenes* OR salutogenic* OR salutogenesis OR generalised resistance resource* OR health asset* OR SOC OR Antonovsky) OR AB (sense of coherence OR Coherence Sense OR Salutogenes* OR salutogenic* OR salutogenesis OR generalised resistance resource* OR health asset* OR SOC OR Antonovsky)  S4 S1 OR S2  S5 S3 AND S4 | 687 |
| Cochrane Library | #1 MeSH descriptor: [Neoplasms] explode all trees  #2 (Tumor* OR Tumour* OR Neoplas* OR Cancer* OR Malignanc* OR oncolog* OR Neoplasm*, Malignant OR Malignant Neoplasm* OR CA):ti,ab,kw  #3 MeSH descriptor: [Carcinoma] explode all trees  #4 (Carcinoma* OR Epithelioma* OR Epithelial Neoplasm*, Malignant OR Malignant Epithelial Neoplasm* OR Neoplasm*, Malignant Epithelial OR Epithelial Tumor*, Malignant OR Malignant Epithelial Tumor* OR Tumor*, Malignant Epithelial OR Carcinoma, Anaplastic OR Anaplastic Carcinoma* OR Carcinoma, Spindle-Cell OR Carcinoma, Spindle Cell OR Spindle-Cell Carcinoma* OR Carcinoma, Undifferentiated OR Undifferentiated Carcinoma*):ti,ab,kw  #5 #1 OR #2 OR #3 OR #4  #6 MeSH descriptor: [Sense of Coherence] explode all trees  #7 (sense of coherence OR Coherence Sense OR Salutogenes* OR salutogenic* OR salutogenesis OR generalised resistance resource* OR health asset* OR SOC OR Antonovsky):ti,ab,kw  #8 #6 OR #7  #9 #5 AND #8 | 2032 |
| CNKI | （篇关摘：心理一致感 + 健康本源论模型 + 有益健康模型 + 抗性资源(精确)) AND（篇关摘：肿瘤 + 癌症 + 恶性肿瘤(精确)) | 79 |
| WANFANG | 主题:("心理一致感" or "健康本源论模型" or "有益健康模型" or "抗性资源") and 主题:("肿瘤" or "癌症" or "恶性肿瘤") | 97 |
| VIP | ((((题名或关键词=心理一致感 OR 题名或关键词=健康本源论模型) OR 题名或关键词=有益健康模型) OR 题名或关键词=抗性资源) AND ((题名或关键词=肿瘤 OR 题名或关键词=癌症) OR 题名或关键词=恶性肿瘤)) | 30 |
| SinoMed | #1 "心理一致感"[加权:扩展]  #2 "心理一致感"[常用字段:智能] OR "健康本源论模型"[常用字段:智能] OR "有益健康模型"[常用字段:智能] OR "抗性资源"[常用字段:智能]  #3 #1 OR #2  #4 "癌症"[常用字段:智能] OR "肿瘤"[常用字段:智能] OR "恶性肿瘤"[常用字段:智能]  #5 #3 AND #4 | 82 |
| *Notes*: CINAHL = Cumulated Index to Nursing and Allied Health Literature. CNKI = China National Knowledge Infrastructure. VIP = China Science and Technology Journal Database. TS = Topics. TI = Title. AB = Abstract. KW = key words. | | |

**Table 2: Characteristics of the included studies.**

| Author-Year,  Country | Study design | SOC  measurement | SOC scores  (*Mean ± SD*) | Cancer type | Participants | | | Correlated factors | | | | | Pearson *r* | | Measurement | |
| --- | --- | --- | --- | --- | --- | --- | --- | --- | --- | --- | --- | --- | --- | --- | --- | --- |
|  |  |  |  |  | Total | Men | Women |  |  |  |  |  |  |  |  |  |
| Henoch I et al. 2007, Sweden(44) | cohort | SOC-13 | —— | Lung cancer | 48 | —— | —— | Quality of life | | | | | 0.298 | | AQEL | |
| Gustavsson-Lilius M  et al. 2007, Finland(10) | cohort | SOC-12（Finnish short version） | —— | Cancer | 123 | 55 | 68 | Sex | | | | | -0.047 | | NA | |
|  |  |  |  |  |  |  |  | Age | | | | | 0.05 | |  |  |
|  |  |  |  |  |  |  |  | Education | | | | | -0.14 | |  |  |
|  |  |  |  |  |  |  |  | Anxiety（Patients） | | | | | -0.56 | | EMAS-State | |
|  |  |  |  |  |  |  |  | Depression（Patients） | | | | | -0.66 | | BDI | |
|  |  |  |  |  |  |  |  | Depression（Partners） | | | | | -0.27 | | BDI | |
|  |  |  |  |  |  |  |  | SOC（Partners） | | | | | 0.24 | | SOC-12 | |
| Henoch I et al. 2010,  Sweden(43) | cross-sectional | SOC-13 | —— | Lung cancer | 106 | 55 | 51 | Quality of life | | | | | 0.50 | | AQEL | |
| Paika V et al. 2010,  Greece(50) | cross-sectional | SOC-29 | 141.5±29.3 | Colorectal cancer | 162 | 109 | 53 | Quality of life | | Physical health | | | 0.450 | | WHOQOL-BREF |  |
|  |  |  |  |  |  |  |  |  |  | Mental health | | | 0.564 | |  |  |
|  |  |  |  |  |  |  |  |  |  | Social relationships | | | 0.375 | |  |  |
|  |  |  |  |  |  |  |  |  |  | Environment | | | 0.536 | |  |  |
| Bonacchi A et al.  2014, Italy(45) | cross-sectional | SOC-3 | —— | Cancer | 764 | —— | —— | Actual Complementary Therapies Users | | | | | 0.102 | | NA |  |
|  |  |  |  |  |  |  |  | Past Complementary Therapies Use | | | | | 0.118 | | NA |  |
| Liu H et al. 2015, China(31) | cross-sectional | SOC-13 | 64. 25±10. 52 | Breast cancer | 232 | —— | 232 | Self - care agency | | | | | 0. 456 | | ESCA |  |
| Shen Q et al. 2016, China(30) | cross-sectional | SOC-13 | 54. 67±5. 12 | Breast cancer | 300 | —— | 300 | Perceived Social Support | | | | | 0. 561 | | PSSS |  |
|  |  |  |  |  |  |  |  | Posttraumatic growth | | | | | 0. 693 | | PTGI |  |
| Tang L L et al. 2017,  China(20) | cross-sectional | SOC-9 | 45.55±9.73 | Breast cancer | 253 | —— | 253 | Quality of life | | | | | 0.371 | | QLQ-C30 |  |
| Leonhart R et al. 2017, China(21) | cross-sectional | SOC-9 | 45.55±9.73 | Breast cancer | 254 | —— | 254 | Somatic symptom severity | | | | | -0.254 | | PHQ-15 |  |
| Jabłoński M J et al. 2019, Poland(48) | cohort | SOC-29 | 141.13±24.17 | Breast cancer | 39 | —— | 39 | Quality of life | | | | | -0.17 | | QLQ-C30 |  |
| Aderhold C et al. 2019, Germany(39) | cross-sectional | SOC-13 | 63.9±11.8 | Cancer | 153 | —— | —— | Depression | | | | Within 1 year since the time of diagnosis | | -0.47 | HADS |  |
|  |  |  |  |  |  |  |  |  |  |  |  | More than 1 year since the time of diagnosis | | -0.51 |  |  |
| von Humboldt S et al. 2019, Portugal(51) | cross-sectional | SOC-29 | The average item score was 3.97±4.12. | Breast cancer | 771 | —— | 771 | Age | | | | | 0.155 | | NA |  |
|  |  |  |  |  |  |  |  | Annual Income | | | | | 0.004 | |  |  |
|  |  |  |  |  |  |  |  | Education | | | | | 0.131 | |  |  |
|  |  |  |  |  |  |  |  | Time since Remission | | | | | 0.535 | |  |  |
|  |  |  |  |  |  |  |  | No limitations and disability | | | | | 0.671 | |  |  |
|  |  |  |  |  |  |  |  | Subjective Well-Being | | | | | 0.144 | | SWLS |  |
|  |  |  |  |  |  |  |  | Adjustment to Aging | | | | | 0.222 | | AtAS |  |
| Bonacchi A et al.  2019, Italy(46) | cross-sectional | SOC-3 | —— | Cancer | 762 | —— | —— | Unmet needs | | | | | -0.220 | | NEQ |  |
| Zhang Q Y et al. 2019, China(22) | cross-sectional | SOC-13 | —— | Breast cancer | 292 | —— | 292 | Acceptance of disability | | | | | 0.375 | | AOD |  |
| Liu H et al. 2019, China(28) | cross-sectional | SOC-13 | 54.21±14.54 | Colorectal cancer | 140 | 88 | 52 | Self - esteem | | | | | 0.489 | | SES |  |
|  |  |  |  |  |  |  |  | Coping | | | Confrontation | | 0.539 | | MCMQ |  |
|  |  |  |  |  |  |  |  |  |  |  | Avoidance | | -0.227 | |  |  |
|  |  |  |  |  |  |  |  |  |  |  | Resignation | | -0.467 | |  |  |
| Ge LN et al. 2019, China(32) | cross-sectional | SOC-13 | 52.96±14.05 | Esophageal cancer | 280 | 225 | 55 | Coping | | | | Positive coping, PC | | 0.552 | SCSQ |  |
|  |  |  |  |  |  |  |  |  |  |  |  | Negative coping, NC | | -0.194 |  |  |
|  |  |  |  |  |  |  |  | General Well - Being | | | | | | 0.486 | GWB |  |
| Krampe H et al. 2020, Germany(41) | cross-sectional | SOC-3 | 12.31±2.59 | Cancer | 945 | 553 | 392 | Mental well-being | | | | | 0.59 | | WHO-5 |  |
|  |  |  |  |  |  |  |  | Perceived hospital and surgery related stress | | | | | -0.42 | | DT |  |
| Asaba K et al. 2021,  Japan(49) | cross-sectional | SOC-13 | 63.28±8.98 | Lung cancer | 66 | 39 | 27 | Sex | | | | | 0.073 | | NA |  |
|  |  |  |  |  |  |  |  | Quality of life | PCS | | | | 0.232 | | SF-8 |  |
|  |  |  |  |  |  |  |  |  | MCS | | | | 0.445 | |  |  |
| Lashani F et al. 2021,  Iran(36) | cross-sectional | SOC-13 | 57.37±17.18 | Breast cancer | 181 | —— | 181 | Sexual function | | | | | 0.20 | | FSFI |  |
|  |  |  |  |  |  |  |  | General well-being | | | | | 0.51 | | HI |  |
| Zamanian H et al.  2021, Iran(37) | cross-sectional | SOC-13 | 57.80±13.47 | Breast cancer | 221 | —— | 221 | Quality of life | | | | | 0.43 | | FACT-B |  |
|  |  |  |  |  |  |  |  | Coping | | | Self-distraction | | 0.16 | | Brief COPE |  |
|  |  |  |  |  |  |  |  |  |  |  | Active coping | | 0.20 | |  |  |
|  |  |  |  |  |  |  |  |  |  |  | denial | | -0.09 | |  |  |
|  |  |  |  |  |  |  |  |  |  |  | substance use | | 0.07 | |  |  |
|  |  |  |  |  |  |  |  |  |  |  | Use of emotional support | | 0.25 | |  |  |
|  |  |  |  |  |  |  |  |  |  |  | Use of instrumental support | | 0.20 | |  |  |
|  |  |  |  |  |  |  |  |  |  |  | Behavioural disengagement | | -0.24 | |  |  |
|  |  |  |  |  |  |  |  |  |  |  | Venting | | -0.14 | |  |  |
|  |  |  |  |  |  |  |  |  |  |  | Positive reframing | | 0.23 | |  |  |
|  |  |  |  |  |  |  |  |  |  |  | Planning | | 0.15 | |  |  |
|  |  |  |  |  |  |  |  |  |  |  | humour | | 0.05 | |  |  |
|  |  |  |  |  |  |  |  |  |  |  | Acceptance | | 0.16 | |  |  |
|  |  |  |  |  |  |  |  |  |  |  | religion | | 0.12 | |  |  |
|  |  |  |  |  |  |  |  |  |  |  | Self-blame | | -0.29 | |  |  |
| Vähäaho N et al. 2021, Finland(42) | cohort | SOC-13 | 70.54±11.93 | Breast cancer | 406 | —— | 406 | Quality of life | | | | | 0.538 | | QLQ-C30 |  |
| Kim H S et al. 2021,  Korea(47) | cross-sectional | SOC-13 | The average item score was 4.86 ± 0.83. | Gynecologic cancer | 148 | —— | 148 | Coping | | | | Positive reframing | | 0.252 | K-CCQ |  |
|  |  |  |  |  |  |  |  |  |  |  |  | Active coping | | 0.100 |  |  |
|  |  |  |  |  |  |  |  |  |  |  |  | Planning | | 0.236 |  |  |
|  |  |  |  |  |  |  |  | Depression | | | | | | -0.541 | HADS |  |
|  |  |  |  |  |  |  |  | Quality of life | | | | | | 0.540 | FACT-G |  |
| Liu H et al. 2021, China(25) | cross-sectional | SOC-13 | 59.82±8.43 | Colorectal cancer | 162 | 98 | 64 | Acceptance of disability | | | | | 0.496 | | ADS-R |  |
|  |  |  |  |  |  |  |  | Psychological distress | | | | | -0.403 | | DT |  |
| Wei M et al. 2021, China(27) | cross-sectional | SOC-13 | 70.93±6.80 | Breast cancer | 852 | —— | 852 | Type C personality | | | | | -0.43 | | CB |  |
|  |  |  |  |  |  |  |  | Depression | | | | | -0.59 | | HAMD |  |
| Zamanian H et al. 2022, Iran(38) | cross-sectional | SOC-13 | 57.80±13.47 | Breast cancer | 221 | —— | 221 | Stigma | | | | | -0.35 | | SSCI-8 |  |
|  |  |  |  |  |  |  |  | Quality of Life | | | | | 0.43 | | FACT-B |  |
| Festerling L et al.  2023, Germany(40) | cross-sectional | SOC-L9 | 32.17 | Cancer | 416 | 138 | 268 | Resilience | | | | | 0.339 | | RS-13 |  |
| Guo Y R et al. 2023, China(23) | cross-sectional | SOC-13 | —— | Breast cancer | 1046 | —— | 1046 | Perceived stress | | | | | -0.625 | | PSS |  |
|  |  |  |  |  |  |  |  | Depression | | | | | -0.850 | | HAMD |  |
| Li Q et al. 2023, China(24) | cross-sectional | SOC-13 | 55.07±10.07 | Cervical cancer | 269 | —— | 269 | Age | | | | | 0.064 | | NA |  |
|  |  |  |  |  |  |  |  | Perceived social support | | | | | 0.424 | | MSPSS |  |
|  |  |  |  |  |  |  |  | Depression | | | | | -0.622 | | CES-D |  |
|  |  |  |  |  |  |  |  | anxiety | | | | | -0.409 | | SAS |  |
| Zheng W et al. 2023, China(9) | cross-sectional | SOC-13 | —— | Lung cancer | 328 | 165 | 163 | Age | | | | | -0.292 | | NA |  |
|  |  |  |  |  |  |  |  | Educational level | | | | | 0.787 | |  |  |
|  |  |  |  |  |  |  |  | Family per capita monthly income | | | | | 0.837 | |  |  |
|  |  |  |  |  |  |  |  | Number of combined chronic diseases | | | | | -0.680 | |  |  |
|  |  |  |  |  |  |  |  | Social alienation | | | | | -0.596 | | GAS |  |
| He L et al. 2023, China(29) | cross-sectional | SOC-13 | 54.17±11.44 | Breast cancer | 240 | —— | 240 | Self-Advocacy | | | | | 0.569 | | FSACS |  |
| Wang RB et al. 2023, China(33) | cross-sectional | SOC-13 | 59.58±10.39 | Lymphoma | 202 | 124 | 78 | The level of resourcefulness | | | | | 0.419 | | RS |  |
|  |  |  |  |  |  |  |  | Family Function | | | | | 0.458 | | FACES II |  |
| Huang TT et al. 2023, China(34) | cross-sectional | SOC-13 | 53.68±20.02 | Thyroid cancer | 200 | —— | 200 | Disease perception | | | | | -0.762 | | BIPQ |  |
| Dadashi N et al. 2024, Iran(35) | cross-sectional | SOC-13 | 60.57±10.77 | Cancer | 200 | 115 | 85 | Death anxiety | | | | | -0.610 | | DAS |  |
| Gu Z H et al. 2024,  China(19) | cross-sectional | SOC-13 | —— | Cervical cancer | 196 | —— | 196 | Severe cancer-related fatigue | | | | | -0.710 | | CFS |  |
| Wang J et al. 2024, China(26) | cross-sectional | SOC-13 | 63.25±11.23 | Malignant tumor | 154 | 86 | 68 | Self - perceived burden | | | | | -0.353 | | SPBS |  |
|  |  |  |  |  |  |  |  | Quality of life | | | | | 0.457 | | SF-8 |  |
| Li H et al. 2025, China(8) | cross-sectional | SOC-13 | 51.20±10.13 | Colorectal cancer | 96 | 51 | 45 | Psychological resilience | | | | | 0.948 | | SRQS |  |
|  |  |  |  |  |  |  |  | Meaning in life | | | | | 0.959 | | MLQ |  |
| *Note:* SOC = Sense of Coherence. NA = Not available. AQEL = Assessment of Quality of Life at the End of Life. EMAS-State = The state-anxiety sub-scale of the Endler Multidimensional Anxiety Scales. BDI = The Beck Depression Inventory. SOC-12 = Sense of Coherence Scale-12 items. WHOQOL-BREF = The World Health Organization Quality of Life Instrument, Short-Form. ESCA = Exercise of Self - care Agency. PSSS = Perceived Social Support Scale. PTGI = Posttraumatic Growth Inventory. QLQ-C30 = European Organization for Research and Treatment of Cancer Questionnaire of Quality of Life. PHQ-15 = Somatic Symptom Severity Scale of the Patient Health Questionnaire. HADS = Hospital Anxiety and Depression Scale. SWLS = The Satisfaction with Life Scale. AtAS = The Adjustment to Aging Scale. NEQ = Needs Evaluation Questionnaire. AOD = Acceptance of disability scale-revised. SES = Rosenberg Self-Esteem Scale. MCMQ = Medical Coping Modes Questionnaire. SCSQ = Simplified Coping Style Questionnaire. GWB = General Well-Being. WHO-5 = The short self-report questionnaire World Health Organization Well-Being Index. DT = The Distress Thermometer. SF-8 = The Short Form-8 Health Survey. FSFI = Female Sexual Function Index. HI = The Health Index. FACT-B = Functional Assessment of Cancer Therapy-Breast scale. Brief COPE = Brief Coping Orientation to Problems Experienced Inventory. K-CCQ = The Korean Form of Cancer Coping Questionnaire. FACT-G = The Functional Assessment of Cancer Therapy-General Scale. ADS-R = The Revised Acceptance of Disability Scale. CB = Cancer behaviors scale. HAMD = Hamilton Depression Rating Scale. SSCI-8 = The stigma scale for chronic illnesses 8-item version. RS-13 = Resilience Scale-13 items. PSS = Perceived Stress Scale. MSPSS = The Multidimensional Scale of Perceived Social Support. CES-D = The Center for Epidemiologic Studies Depression Scale. SAS = The Zung Self-Rating Anxiety Scale. GAS = General Alienation Scale. FSACS = Female Self-Advocacy in Cancer Survivorship. RS = Resourcefulness Scale. FACES II = Family Adaptation and Cohesion Evaluation Scale II. BIPQ = The Brief Illness Perception Questionnaire. DAS = Templer’ s Death Anxiety Scale. CFS = The Cancer Fatigue Scale. SPBS = Self-Perceived Burden Scale. SRQS = Stress Resilience Quotient Scale. MLQ = Meaning in Life Questionnaire. | | | | | | | | | | | | | | | |  |

**Table 3: Quality assessment of cross-sectional studies.**

| Studies | Item 1 | Item 2 | Item 3 | Item 4 | Item 5 | Item 6 | Item 7 | Item 8 | Item 9 | Item10 | Item11 | Total score | Quality grade |
| --- | --- | --- | --- | --- | --- | --- | --- | --- | --- | --- | --- | --- | --- |
| Henoch I et al. 2010,  Sweden(43) | Yes | No | Yes | Yes | No | No | No | No | No | Yes | No | 4 | Moderate |
| Paika V et al. 2010,  Greece(50) | Yes | No | Yes | Yes | No | No | No | Yes | No | Yes | No | 5 | Moderate |
| Bonacchi A et al.  2014, Italy(45) | Yes | No | No | Yes | No | No | Yes | Yes | No | No | No | 4 | Moderate |
| Liu H et al. 2015, China(31) | Yes | No | Yes | Yes | No | No | No | Yes | No | No | No | 4 | Moderate |
| Shen Q et al. 2016, China(30) | Yes | No | Yes | Yes | No | Yes | No | No | No | No | No | 4 | Moderate |
| Tang L L et al. 2017,  China(20) | Yes | No | Yes | Yes | No | Yes | No | No | No | Yes | No | 5 | Moderate |
| Leonhart R et al. 2017, China(21) | Yes | No | Yes | Yes | No | Yes | No | Yes | No | Yes | No | 6 | Moderate |
| Aderhold C et al. 2019, Germany(39) | Yes | No | Yes | Yes | No | No | Yes | Yes | Yes | Yes | No | 7 | Moderate |
| von Humboldt S et al. 2019, Portugal(51) | Yes | No | Yes | Yes | No | Yes | Yes | Yes | Yes | No | No | 7 | Moderate |
| Bonacchi A et al.  2019, Italy(46) | Yes | No | Yes | Yes | No | Yes | Yes | No | No | No | No | 5 | Moderate |
| Zhang Q Y et al. 2019, China(22) | Yes | No | Yes | No | No | Yes | Yes | Yes | No | Yes | No | 6 | Moderate |
| Liu H et al. 2019, China(28) | Yes | No | Yes | Yes | No | Yes | No | No | No | No | No | 4 | Moderate |
| Ge LN et al. 2019, China(32) | Yes | No | Yes | Yes | No | No | No | Yes | No | No | No | 4 | Moderate |
| Krampe H et al. 2020, Germany(41) | Yes | No | Yes | Yes | No | No | Yes | Yes | No | No | No | 5 | Moderate |
| Asaba K et al. 2021,  Japan(49) | Yes | No | Yes | Yes | No | No | No | Yes | No | Yes | No | 5 | Moderate |
| Lashani F et al. 2021,  Iran(36) | Yes | No | Yes | Yes | Yes | Yes | Yes | Yes | No | Yes | No | 8 | High |
| Zamanian H et al.  2021, Iran(37) | Yes | No | Yes | Yes | No | Yes | Yes | Yes | No | Yes | No | 7 | Moderate |
| Kim H S et al. 2021,  Korea(47) | Yes | No | Yes | Yes | No | Yes | Yes | Yes | No | Yes | No | 7 | Moderate |
| Liu H et al. 2021, China(25) | Yes | No | Yes | Yes | No | Yes | No | No | No | No | No | 4 | Moderate |
| Wei M et al. 2021, China(27) | Yes | No | Yes | No | No | Yes | No | Yes | No | No | No | 4 | Moderate |
| Zamanian H et al. 2022, Iran(38) | Yes | No | Yes | Yes | No | No | Yes | Yes | No | Yes | No | 6 | Moderate |
| Festerling L et al.  2023, Germany(40) | Yes | No | Yes | Yes | No | No | Yes | No | Yes | Yes | No | 6 | Moderate |
| Guo Y R et al. 2023, China(23) | Yes | No | Yes | Yes | No | Yes | No | Yes | No | No | No | 5 | Moderate |
| Li Q et al. 2023, China(24) | Yes | No | Yes | Yes | No | No | No | Yes | No | Yes | No | 5 | Moderate |
| Zheng W et al. 2023, China(9) | Yes | No | Yes | Yes | No | Yes | No | Yes | No | No | No | 5 | Moderate |
| He L et al. 2023, China(29) | Yes | No | Yes | Yes | No | Yes | No | Yes | No | No | No | 5 | Moderate |
| Wang RB et al. 2023, China(33) | Yes | No | Yes | Yes | No | Yes | No | No | No | No | No | 4 | Moderate |
| Huang TT et al. 2023, China(34) | Yes | No | Yes | No | No | Yes | No | Yes | No | No | No | 4 | Moderate |
| Dadashi N et al. 2024, Iran(35) | Yes | No | Yes | Yes | No | Yes | Yes | Yes | No | No | No | 6 | Moderate |
| Gu Z H et al. 2024,  China(19) | Yes | No | Yes | Yes | No | No | No | Yes | Yes | No | No | 5 | Moderate |
| Wang J et al. 2024, China(26) | Yes | No | Yes | Yes | No | Yes | No | No | Yes | No | No | 5 | Moderate |
| Li H et al. 2025, China(8) | Yes | No | Yes | No | No | Yes | No | Yes | No | No | No | 4 | Moderate |

**Table 4: Quality assessment of cohort studies.**

| Studies | Selection  (0~4 stars) | | | | Comparability  (0~2 stars) | Exposure  (0~3 stars) | | | Total score | Quality grade |
| --- | --- | --- | --- | --- | --- | --- | --- | --- | --- | --- |
|  | Item 1 | Item 2 | Item 3 | Item 4 | Item 5 | Item 6 | Item 7 | Item 8 |  |  |
| Henoch I et al. 2007, Sweden(44) | * | * |  |  |  |  | * | * | 4 | Moderate |
| Gustavsson-Lilius M  et al. 2007, Finland(10) | * | * |  |  |  |  | * | * | 4 | Moderate |
| Jabłoński M J et al. 2019, Poland(48) | * | * |  |  |  |  | * | * | 4 | Moderate |
| Vähäaho N et al. 2021, Finland(42) | * | * |  |  |  |  | * | * | 4 | Moderate |
| Notes: Item 1: Representativeness of the exposed cohort. Item 2: Selection of the non exposed cohort. Item 3: Ascertainment of exposure. Item 4: Demonstration that outcome of interest was not present at start of study. Item 5: Comparability of cohorts on the basis of the design or analysis. Item 6: Assessment of outcome . Item 7: Was follow-up long enough for outcomes to occur. Item 8: Adequacy of follow up of cohorts. | | | | | | | | | | |

**Figure 1-11: Factors associated with sense of coherence in patients with malignant tumors: Forest plots.**


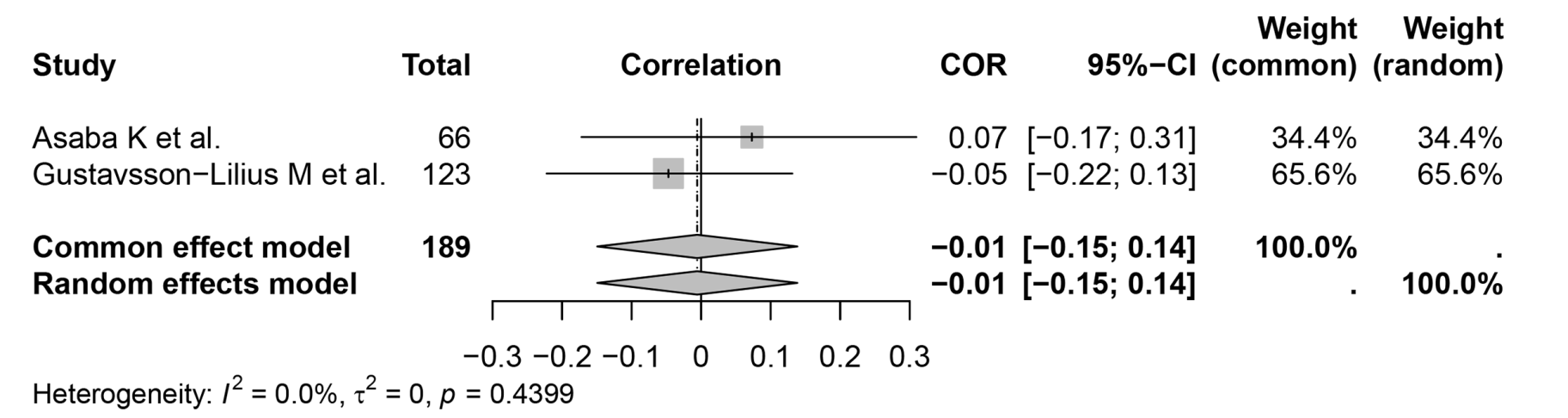


**Figure1. Forest plot of the correlation between gender and sense of coherence**


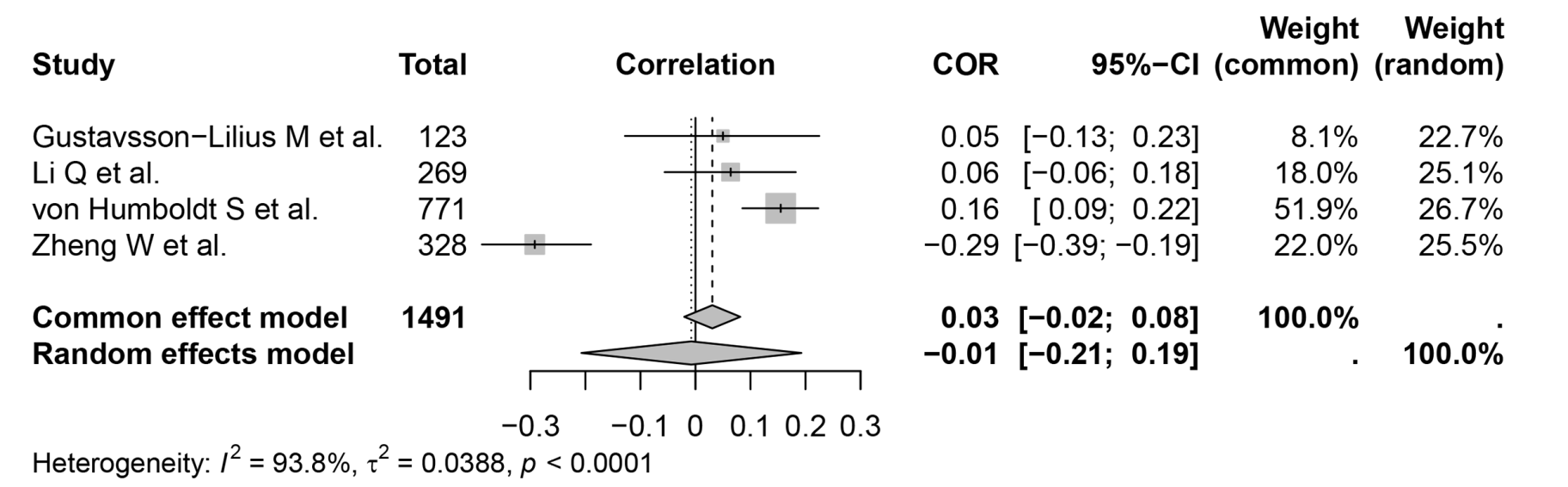


**Figure 2. Forest plot of the correlation between age and sense of coherence**


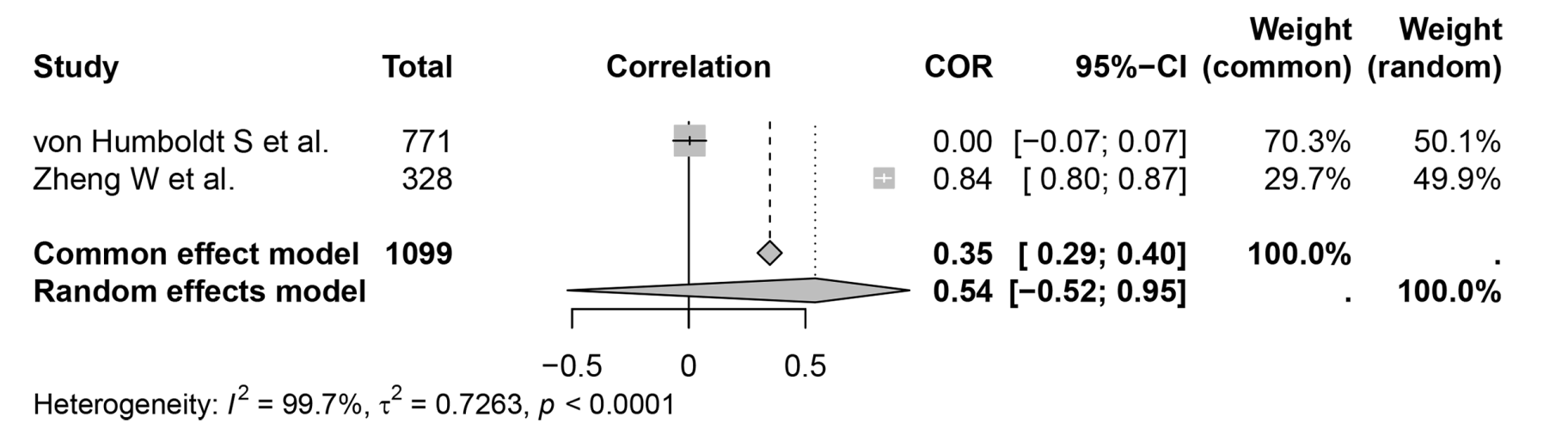


**Figure 3. Forest plot of the correlation between income and sense of coherence**


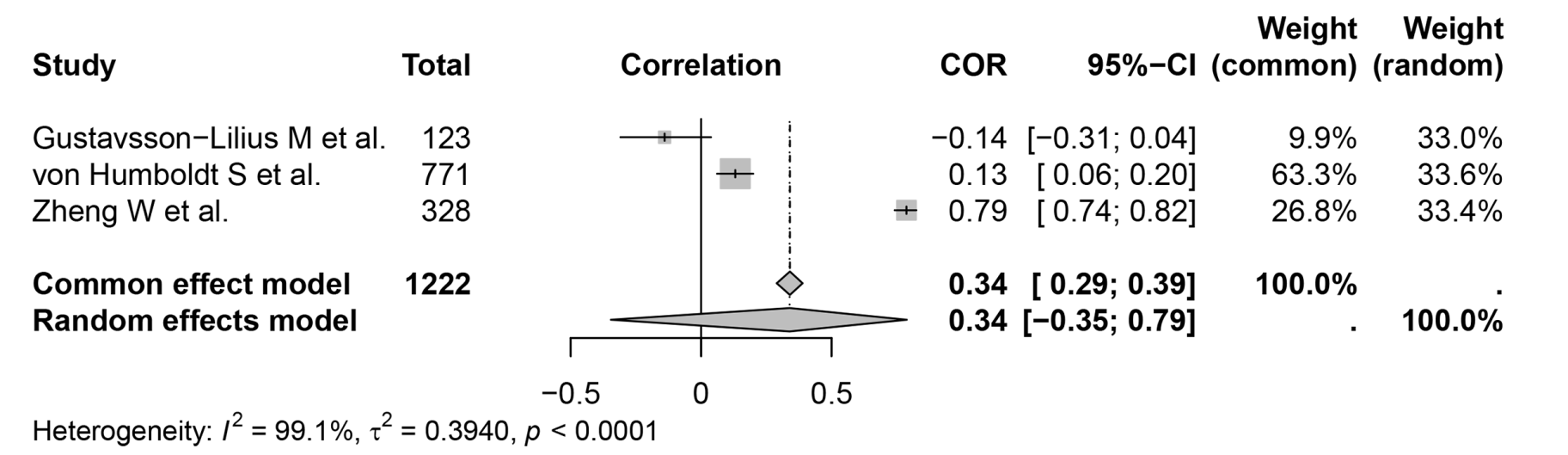


**Figure 4. Forest plot of the correlation between educational level and sense of coherence**


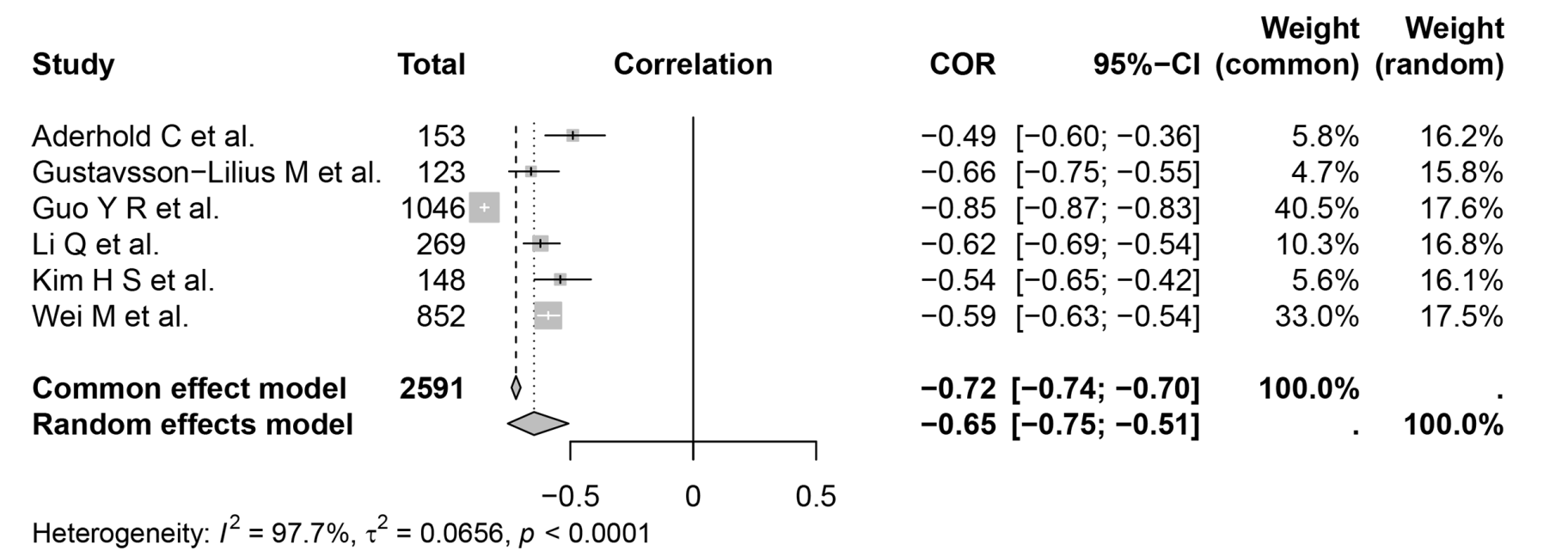


**Figure 5. Forest plot of the correlation between depression and sense of coherence**


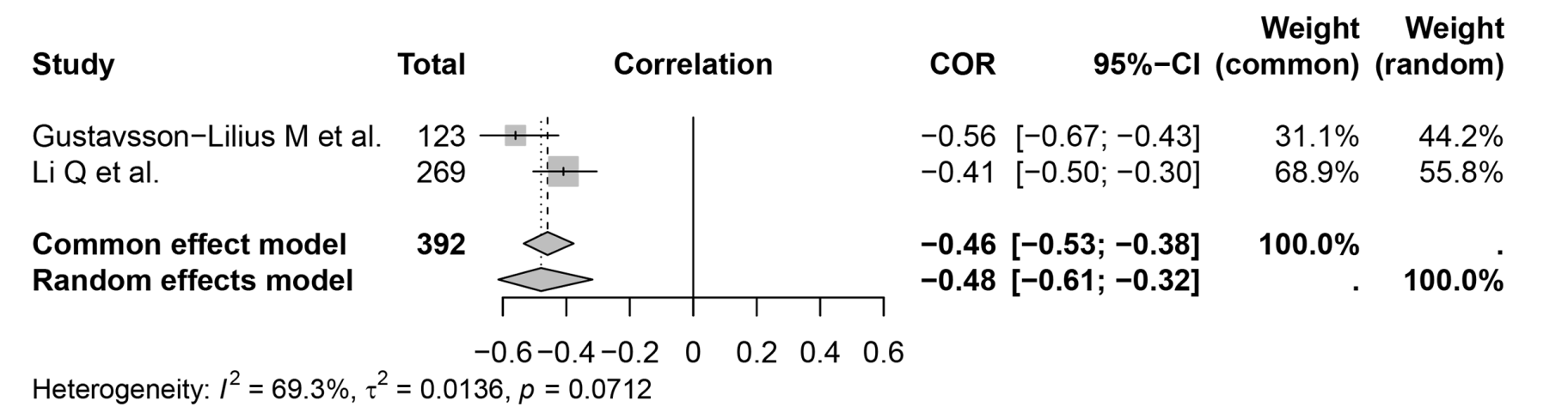


**Figure 6. Forest plot of the correlation between anxiety and sense of coherence**

**
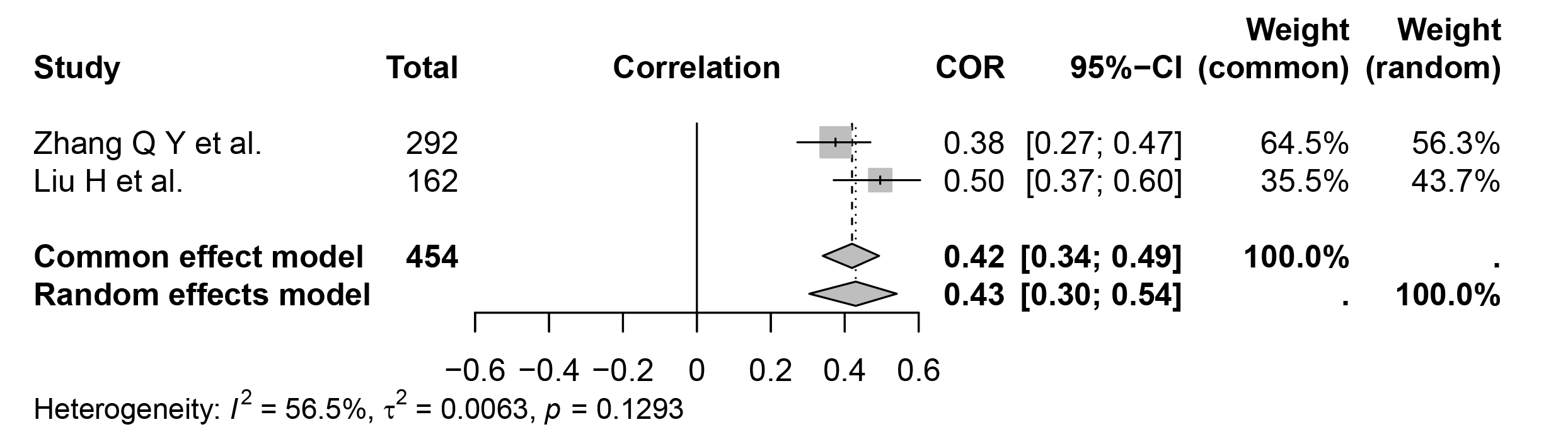
**

**Figure 7. Forest plot of the correlation between acceptance of disability and sense of coherence**


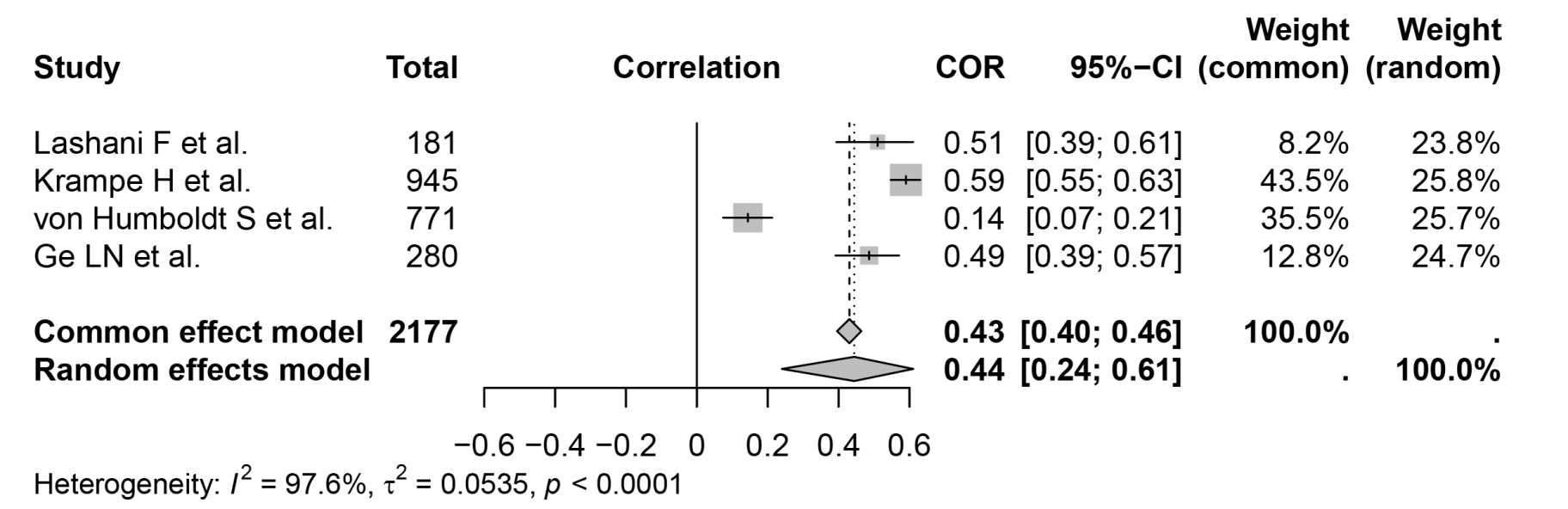


**Figure 8. Forest plot of the correlation between well-being and sense of coherence**


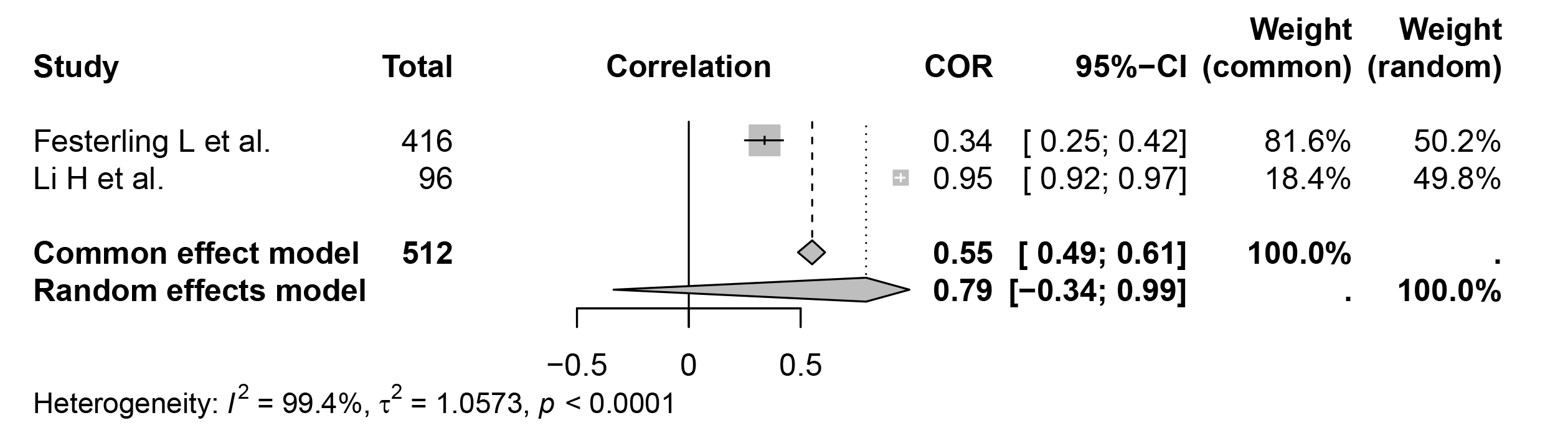


**Figure 9. Forest plot of the correlation between resilience and sense of coherence**


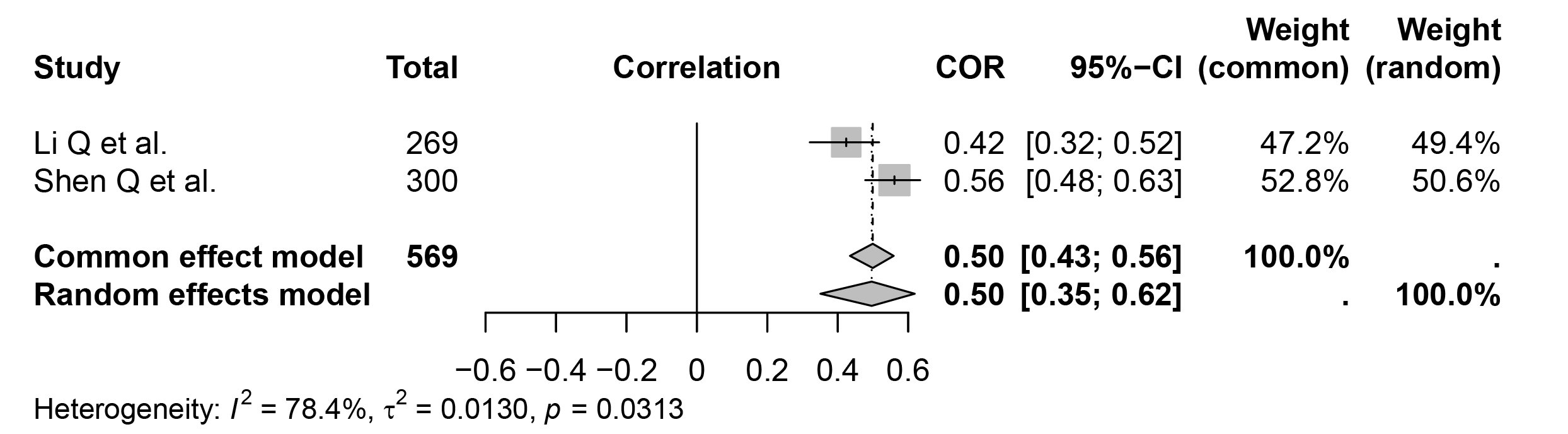


**Figure 10. Forest plot of the correlation between perceived social support and sense of coherence**


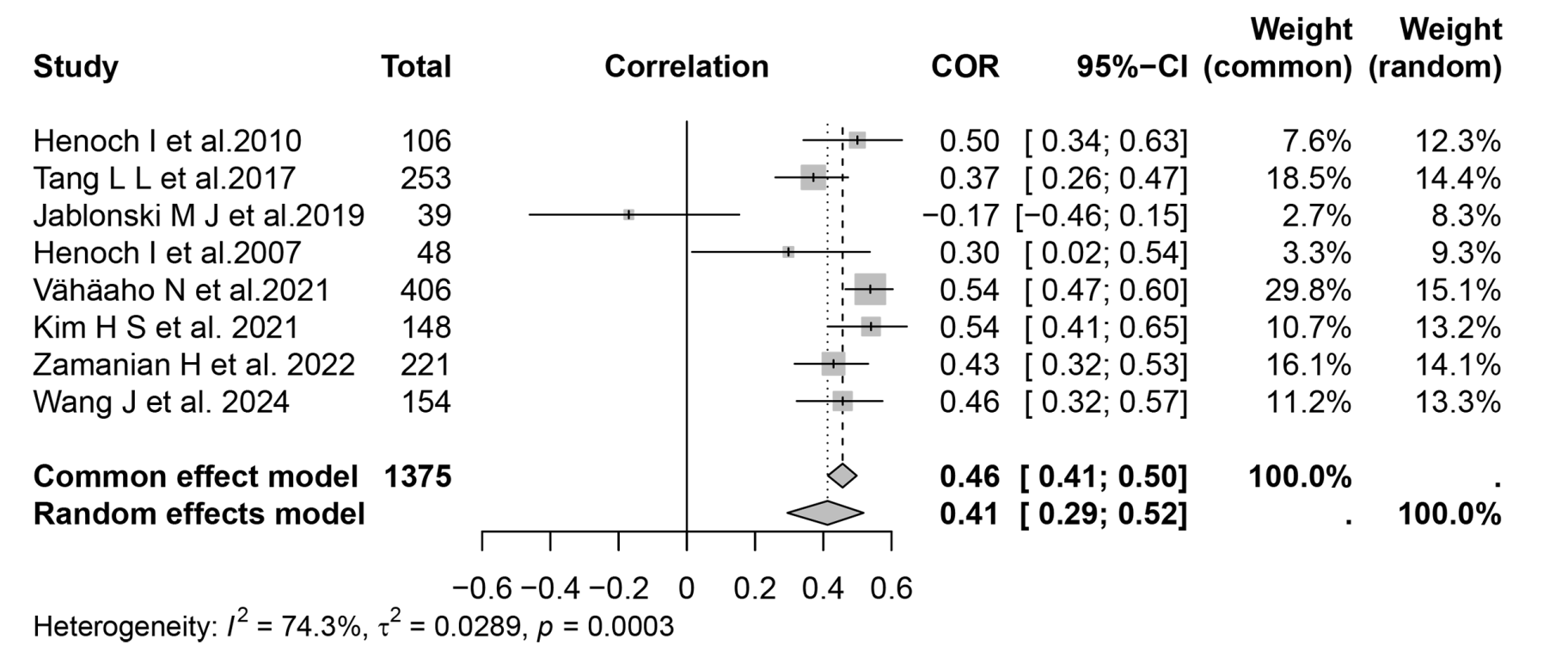


**Figure 11. Forest plot of the correlation between quality of life and sense of coherence**

**Figure 12-16: Factors associated with sense of coherence in patients with malignant tumors: Sensitivity analysis.**


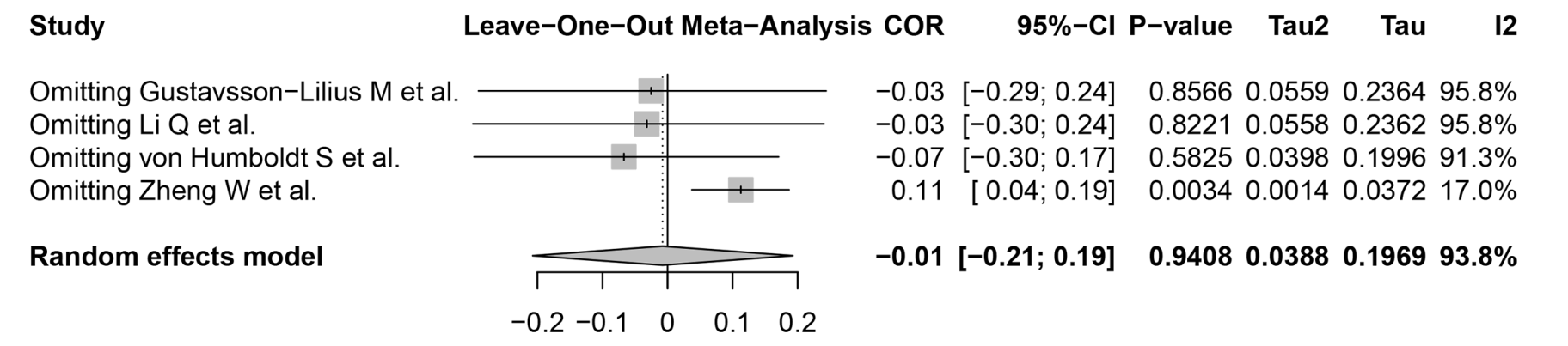


**Figure 12. Sensitivity analysis of the correlation between age and sense of coherence**


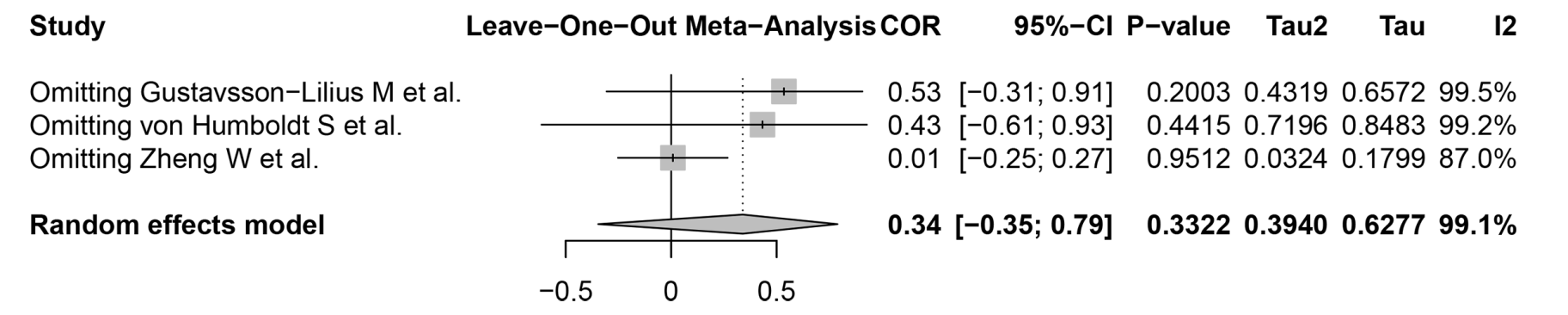


**Figure 13. Sensitivity analysis of the correlation between educational level and sense of coherence**


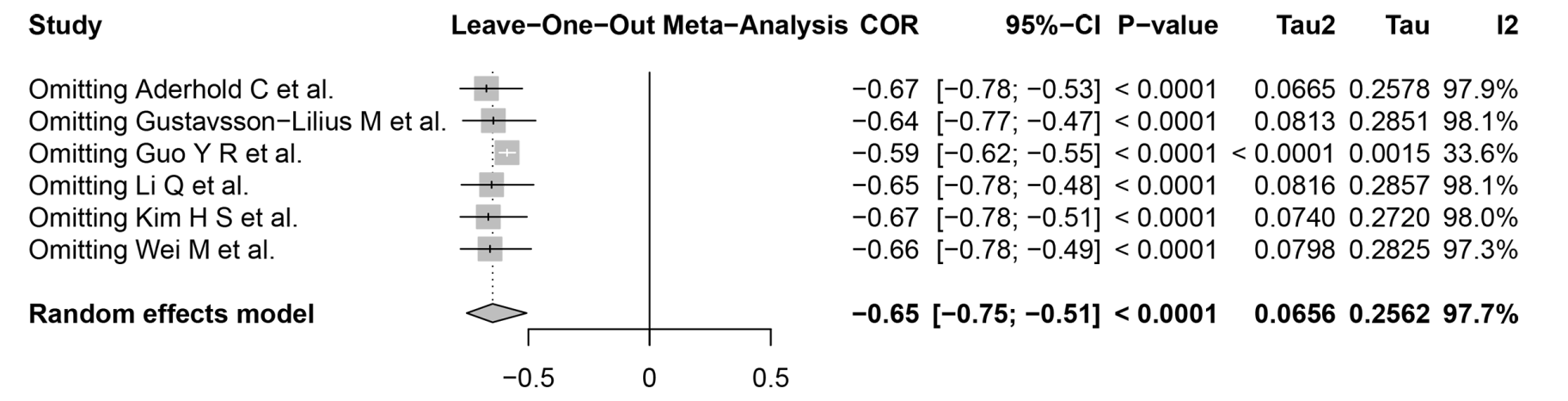


**Figure 14. Sensitivity analysis of the correlation between depression and sense of coherence**


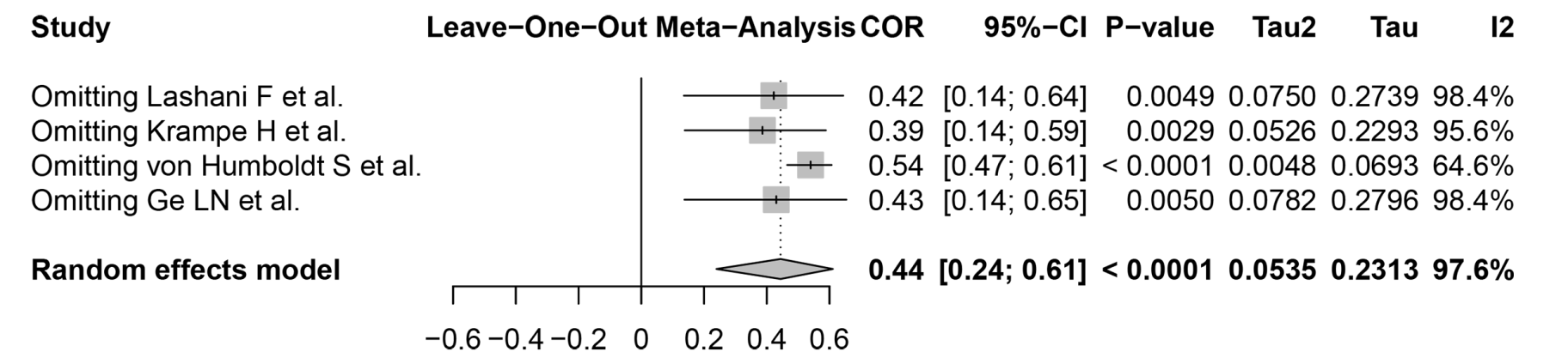


**Figure 15. Sensitivity analysis of the correlation between well-being and sense of coherence**


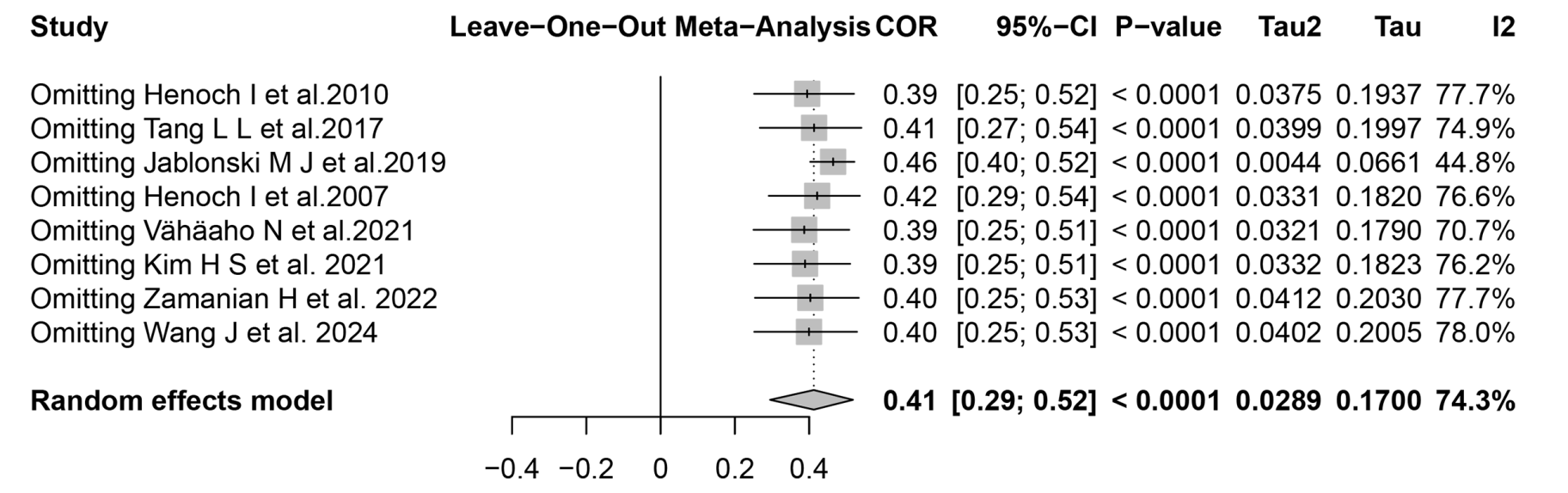


**Figure 16. Sensitivity analysis of the correlation between quality of life and sense of coherence**

**Table 5: Subgroup analysis of the correlations for sense of coherence.**

| Correlated factors | Subgroups | | Number | Sample | Heterogeneity test | | *r* | 95%*CI* | *P* |
| --- | --- | --- | --- | --- | --- | --- | --- | --- | --- |
|  |  |  |  |  | *I*^2^ (%) | *P* |  |  |  |
| Age | Measurement tool for SOC | SOC-12（Finnish short version） | 1 | 123 | NA | NA | 0.05 | -0.13, 0.23 | 0.2066 |
|  |  | SOC-13 | 2 | 597 | 94.9 | ＜0.0001 | -0.12 | -0.44, 0.23 |  |
|  |  | SOC-29 | 1 | 771 | NA | NA | 0.16 | 0.09, 0.22 |  |
|  | Region | Finland | 1 | 123 | NA | NA | 0.05 | -0.13, 0.23 | 0.2066 |
|  |  | China | 2 | 597 | 94.9 | ＜0.0001 | -0.12 | -0.44, 0.23 |  |
|  |  | Portugal | 1 | 771 | NA | NA | 0.16 | 0.09, 0.22 |  |
| Depression | Cancer | Unclear | 2 | 276 | 77.2 | 0.0360 | -0.58 | -0.72, -0.39 | 0.4954 |
|  |  | Breast cancer | 2 | 1898 | 99.4 | ＜0.0001 | -0.75 | -0.91, -0.38 |  |
|  |  | Cervical cancer | 1 | 269 | NA | NA | -0.62 | -0.69, -0.54 |  |
|  |  | Gynecologic cancer | 1 | 148 | NA | NA | -0.54 | -0.65, -0.42 |  |
|  | Measurement tool for SOC | SOC-13 | 5 | 2468 | 98.1 | ＜0.0001 | -0.64 | -0.79, -0.42 | 0.8776 |
|  |  | SOC-12（Finnish short version） | 1 | 123 | NA | NA | -0.66 | -0.75, -0.55 |  |
|  | Region | Germany | 1 | 153 | NA | NA | -0.49 | -0.60, -0.36 | 0.1145 |
|  |  | Finland | 1 | 123 | NA | NA | -0.66 | -0.75, -0.55 |  |
|  |  | China | 3 | 2167 | 98.8 | ＜0.0001 | -0.71 | -0.86, -0.44 |  |
|  |  | Korea | 1 | 148 | NA | NA | -0.54 | -0.65, -0.42 |  |
|  | Measurement tool for depression | HADS | 2 | 301 | 0 | 0.5506 | -0.52 | -0.59, -0.43 | 0.0829 |
|  |  | BDI | 1 | 123 | NA | NA | -0.66 | -0.75, -0.55 |  |
|  |  | HAMD | 2 | 1898 | 99.4 | ＜0.0001 | -0.75 | -0.91, -0.38 |  |
|  |  | CES-D | 1 | 269 | NA | NA | -0.62 | -0.69, -0.54 |  |
| Well-being | Cancer | Unclear | 1 | 945 | NA | NA | 0.59 | 0.55, 0.63 | 0.0372 |
|  |  | Breast cancer | 2 | 952 | 96.0 | ＜0.0001 | 0.34 | -0.06, 0.64 |  |
|  |  | Esophageal cancer | 1 | 280 | NA | NA | 0.49 | 0.39, 0.57 |  |
|  | Measurement tool for SOC | SOC-13 | 2 | 461 | 0 | 0.7397 | 0.50 | 0.42, 0.56 | <0.0001 |
|  |  | SOC-3 | 1 | 945 | NA | NA | 0.59 | 0.55, 0.63 |  |
|  |  | SOC-29 | 1 | 771 | NA | NA | 0.14 | 0.07, 0.21 |  |
| Quality of life | Cancer | Lung cancer | 2 | 154 | 45.5 | 0.1757 | 0.43 | 0.22, 0.60 | 0.3555 |
|  |  | Breast cancer | 4 | 919 | 87.2 | ＜0.0001 | 0.36 | 0.17, 0.52 |  |
|  |  | Gynecologic cancer | 1 | 148 | NA | NA | 0.54 | 0.41, 0.65 |  |
|  |  | Unclear | 1 | 154 | NA | NA | 0.46 | 0.32, 0.57 |  |
|  | Measurement tool for SOC | SOC-13 | 6 | 1083 | 20.4 | 0.2796 | 0.49 | 0.43, 0.54 | < 0.0001 |
|  |  | SOC-9 | 1 | 253 | NA | NA | 0.37 | 0.26, 0.47 |  |
|  |  | SOC-29 | 1 | 39 | NA | NA | -0.17 | -0.46, 0.15 |  |
|  | Region | Sweden | 2 | 154 | 45.5 | 0.1757 | 0.43 | 0.22, 0.60 | 0.0002 |
|  |  | China | 2 | 407 | 1.7 | 0.3133 | 0.40 | 0.32, 0.48 |  |
|  |  | Poland | 1 | 39 | NA | NA | -0.17 | -0.46, 0.15 |  |
|  |  | Finland | 1 | 406 | NA | NA | 0.54 | 0.47, 0.60 |  |
|  |  | Korea | 1 | 148 | NA | NA | 0.54 | 0.41, 0.65 |  |
|  |  | Iran | 1 | 221 | NA | NA | 0.43 | 0.32, 0.53 |  |
|  | Measurement tool for QOL | AQEL | 2 | 154 | 45.5 | 0.1757 | 0.43 | 0.22, 0.60 | 0.4874 |
|  |  | QLQ-C30 | 3 | 698 | 91.4 | ＜0.0001 | 0.31 | 0.02, 0.55 |  |
|  |  | FACT-G | 1 | 148 | NA | NA | 0.54 | 0.41, 0.65 |  |
|  |  | FACT-B | 1 | 221 | NA | NA | 0.43 | 0.32, 0.53 |  |
|  |  | SF-8 | 1 | 154 | NA | NA | 0.46 | 0.32, 0.57 |  |
